# Genome-Wide Association Study of Root and Shoot Related Traits in Spring Soybean (*Glycine max* L.) at Seedling Stages Using SLAF-Seq

**DOI:** 10.3389/fpls.2021.568995

**Published:** 2021-07-28

**Authors:** Ajmal Mandozai, Abdourazak Alio Moussa, Qi Zhang, Jing Qu, Yeyao Du, Gulaqa Anwari, Noor Al Amin, Piwu Wang

**Affiliations:** College of Agronomy, Plant Biotechnology Center, Jilin Agricultural University, Changchun, China

**Keywords:** GWAS, soybean, SLAF-seq, root and shoot related traits, SNPs, genes, qRT-PCR

## Abstract

Root systems can display variable genetic architectures leading to nutrient foraging or improving abiotic stress tolerance. Breeding for new soybean varieties with efficient root systems has tremendous potential in enhancing resource use efficiency and plant adaptation for challenging climates. In this study, root related traits were analyzed in a panel of 260 spring soybean with genome-wide association study (GWAS). Genotyping was done with specific locus amplified fragment sequencing (SLAF-seq), and five GWAS models (GLM, MLM, CMLM, FaST-LMM, and EMMAX) were used for analysis. A total of 179,960 highly consistent SNP markers distributed over the entire genome with an inter-marker distance of 2.36 kb was used for GWAS analysis. Overall, 27 significant SNPs with a phenotypic contribution ranging from 20 to 72% and distributed on chromosomes 2, 6, 8, 9, 13, 16 and 18 were identified and two of them were found to be associated with multiple root-related traits. Based on the linkage disequilibrium (LD) distance of 9.5 kb for the different chromosomes, 11 root and shoot regulating genes were detected based on LD region of a maximum 55-bp and phenotypic contribution greater than 22%. Expression analysis revealed an association between expression levels of those genes and the degree of root branching number. The current study provides new insights into the genetic architecture of soybean roots, and the underlying SNPs/genes could be critical for future breeding of high-efficient root system in soybean.

## Introduction

Soybean (*Glycine max* L.) is the third worldwide cultivated crop that plays a significant role in food and industrial production (Tran and Mochida, 2010). Globally, the human population is projected to rise up to 25% and nearly hit ten billion. Still, the current rate of genetic yield in key crops species is deficient to fulfill upcoming demand (Hickey et al., 2019). The lack of water, lodging and deficiency of nutrients induced by extreme climate will seriously reduce crop production and endanger worldwide food safety (Sinclair, 2011). Soybean is a major oilseed crop in China, growing on around eight million hectares (Zhang et al., 2004); therefore, continuous improvement in soybean quality and yield is vital.

The soybean roots grow from the seed radical, which has three distinct morphologically established components: a primary taproot that drills down into the soil and originates as a radicle from a germinating seed which holds the plant in place; fibrous (lateral) roots that spread out throughout the soil, also referred to as secondary roots that emerge from the taproot, and tertiary roots that originate from lateral roots and take up the nutrients and water from soil (Carlson and Lersten, 2004). Over the last century, most soybean breeding efforts have concentrated on the invention and development of aboveground characters such as, end-use quality and yield (Assefa et al., 2018).

Roots, the below ground part of the plant which is obscured from direct observation, received nearly no direct thought for selection and breeding. Thus, root characteristics are rarely considered as selection criteria (Tuberosa and Salvi, 2007), and therefore fewer root related genes have been detected and many genes leading to root development remain unknown in soybean. However, indirect selection via secondary phenotypes, for example nutrient uptake and drought tolerance was used as a proxy to improve root systems (Rosa et al., 2019). Roots play an important role in absorbing water and nutrients as well as providing protection against biotic and abiotic stresses that can directly affect plant growth, above-ground development and final yield (Khan et al., 2016). In crops, root and shoot characteristics are related to environmental stresses like salinity, wind, aluminum toxicity, flood and drought (Keller et al., 1999; Liu et al., 2005b). A well-developed root system is one of the crop protection mechanisms to defend against abiotic stress. Because to their relative stability across different environments, many root related quantitative traits, such as root biomass, root length, and root volume, usually exhibited high heritability (Tian, 1984; Pantalone et al., 1996; Liu et al., 2005b). Besides, the root system is influenced by management, environmental conditions, and is genotype-dependent as plants respond to soil nutrient restriction and water shortage by increasing root biomass and thus increasing the ratio of root to shoot biomass (Bloom et al., 1985; Abdel-Ghani et al., 2013). Moreover, since roots provide the interface between plants and the complex soil environment (Smith and De Smet, 2012), it is advisable to use root improvement as the main purpose of soybean resistance breeding (such as drought, storm, flood, aluminum toxicity, etc.) (Rong et al., 2011).

Due to the challenge in achieving reliable root trait data from the field, characterizing crops like soybean with improved root system characteristics in the field remains still a major defy to current plant biology and, root phenotyping studies are commonly conducted under controlled environments (Rong et al., 2011; Seck et al., 2020; Chen et al., 2021). Besides, some pieces of evidence support that high yielding soybean varieties are supposed to have propitious root systems at the early stages, which may strongly sustain water and nutrients, resulting in increased yield especially under limited water or nutrient availability (Seck et al., 2020; Chen et al., 2021; Dayoub et al., 2021). To date, several Quantitative Trait Loci (QTL) studies have been conducted to locate shoot and root-related trait QTLs in soybean under various conditions of growth, at diverse developmental stages and involving various genetic populations (Rong et al., 2011; Brensha et al., 2012; Manavalan et al., 2015; Prince et al., 2020; Seck et al., 2020; Chen et al., 2021). Rong et al. (2011) in a controlled experiment under hydroponic culture at seedling stage, used a mapping population containing 165 soybean recombinant inbred lines (RILs) derived from the cross between Zhongdou29 and Zhongdou32, and detected 20 QTLs associated with various root and shoot traits located on eight different chromosomes, along with 9 major QTLs on chromosomes 11 and 14. Brensha et al. (2012) in a QTL analysis of root and shoot traits, utilized a RIL population originated from two contrasted parental lines, ‘Essex’ and ‘Forrest’, and mapped 12 QTLs distributed on chromosomes 1, 3, 6, 8, 13, 14, and 18. Those QTLs were associated with multiple root and shoot traits including basal root thickness, lateral root number, maximum root length, root fresh weight (RFW), root dry weight (RDW), shoot fresh weight (SFW), shoot dry weight (SDW), and RFW/SFW, and RDW/SDW ratios. Manavalan et al. (2015) in a study involving 251 BC2F5 backcross inbred lines developed from the cross Dunbar/PI 326582A, and using a well-watered cone system in a growth chamber, identified a major QTL on chromosome 8 (*Satt315-I locus*) controlling various seedling shoot and root traits including tap root length, lateral root number, shoot length, and shoot dry weight. Recently, using an inter-specific mapping population comprising 184 F_7:8_ RILs grown in a cone system, Prince et al. (2020) constructed a high resolution genetic linkage map, and identified 11 putative QTLs associated with several root and shoot traits at V1 stage of soybean growth. The detected QTLs involved five chromosomes (chromosomes 3, 7, 8, 14, and 20) and harbored various candidate genes. Lately, in a trial using 3-weeks old seedlings derived from a cross between K099 and Fendou16 grown under hydroponic conditions, Chen et al. (2021) successfully detected and validated a major QTL associated with primary root length trait located on chromosome 16 between Sat_165 and Satt621 interval markers. Yet, due to low-density markers and large confidence intervals, the localization of these different QTLs was inconsistent among the different findings and further soybean root studies were necessary to detect more chromosomal regions and ultimately identify consistent loci to further screen and identify candidate genes crucial for marker-assisted selection.

Genome-wide association study (GWAS) is an influential technique that helps researchers to determine that polymorphisms are associated with a specific trait in hundreds or thousands of individuals in the genome, enabling repeated SNPs to be checked against specific traits. Today, the latest progress in sequencing technologies and bioinformatics approaches has rendered GWAS an efficient way compared to conventional QTL mapping methods (Thomson, 2014). GWAS is commonly used in recent years to examine the genetic basis of complex traits in main crops such as soybean and rice (Zhao et al., 2011; Zhou et al., 2015; Huang et al., 2016). Prince et al. (2019) discovered many SNPs and candidate genes associated with multiple seedling root architectural traits, including lateral root number, root surface area, and root volume, using a genome-wide association study involving 397 soybean accessions. In a comprehensive genome-wide association analysis involving a panel of 137 early maturing soybean lines genotyped via genotyping-by-sequencing and whole-genome sequencing, Seck et al. (2020) characterized twelve root system architecture traits and revealed 10 SNPs explaining 15 to 25% of the phenotypic variation for root total length and primary root diameter. Those QTL regions harbored chromosomes 1, 3, 6, 7, 10, 13, 18, and 19, and contained two putative candidate genes. Rapid progress in Next-Generation Sequencing (NGS) relevant technologies, such as Specific-Locus Amplified Fragment sequencing (SLAF-seq) has generated a huge number of SNPs that provide substantial opportunities for GWAS studies for various traits in different crops (Hake et al., 2017; Pandey et al., 2017; Zhang X. et al., 2017; Zheng et al., 2018). Furthermore, SLAF-seq was proposed as a fast, reliable, highly efficient and cost-effective method to develop large-scale SNP (Sun et al., 2013) and used in many crops like rice, sesame, cucumber, *Brassica napus*, etc. (Zhang et al., 2013; Xu et al., 2015; Geng et al., 2016; Li Y. et al., 2017). However, to our knowledge, there is no available report about genome-wide studies for root and shoot related traits using SLAF-seq in soybean. In this study, a panel of 260 spring soybean was used to conduct GWAS using SLAF-seq technology to investigate the genetic basis underlying soybean root and shoot related traits at VC, V1 and V2 stages.

The present study aims to (i) examine phenotypic variation of root and shoot related traits in the spring soybean germplasm; (ii) perform GWAS to detect candidate genes and SNPs associated with root and shoot related traits; and (iii) check the expression level of potential candidate genes associated with root and shoot development across stages.

## Materials and Methods

### Plant Material and Experimental Design

In this study, a panel of 260 spring soybean germplasm was used, collected from different sites that cover the major distribution areas in China (Supplementary File 1). A greenhouse soil experiment was arranged in a completely randomized design (CRD) with three replications at the department of Agronomy at Jilin Agricultural University (43.8139° N, 125.4066° E). The settings for the greenhouse were: 28/24°C temperature, 65/85% relative humidity, and 16/8 h photoperiod day/night, respectively. The natural population seeds were directly seeded in polyvinyl chloride (PVC) pipes (8 cm in diameter and 3.2 mm thick, 25 cm height) containing a mixture of soil and vermiculite (2:1), and regularly watered at 5-day intervals in spring 2019.

### Phenotypic Evaluation and Root Scanning

The root and shoot traits were investigated in three vegetative stages VC (after cotyledons and unifoliate leaves have fully Expanded), V1 (Fully developed leaves at unifoliolate nodes) and V2 (Fully developed trifoliate leaf at node above the unifoliolate nodes) (Fehr and Caviness, 1977) corresponding to 7, 14, and 21 days after germination, respectively. Root growth in soybean is well known to show linear growth from the vegetative (V1) to mid-reproductive (R5) growth stages (Rong et al., 2011). The experiments were repeated three times to increase the credibility of root related traits measurements. The seedlings were carefully removed from soil at the specific time-points and rinsed with tap water to remove the soil. The procedure of washing root was conducted carefully to avoid supplementary root injury and losses. Manually evaluated traits were shoot length (SHL), shoot dry weight (SDW), root dry weight (RDW), total plant biomass (TPB), and root dry weight to shoot dry weight ratio (RDW/SDW) (Table 1). Shoot length was manually measured with the help of a ruler from the top tip of the primary leaf to the base of shoot. After shoot measuring each root system was cut from the shoot and roots were individually scanned via a root scanner-based image (Perfection V800; Epson) set to a resolution of 12800 dots per inch (dpi: 5039.37 dots per cm) then analyzed using DJ-GX02 software^1^ (Supplementary File 2). Five root morphological traits were recorded using the root analysis software (Table 1). If data collection could not be measured on the same day, seedlings were conserved by submerging roots in 75% ethanol to avoid further growth of seedlings. For dry weight biomass, root and shoot were collected individually and dried in the oven for 48 h at 75°C, to achieve the constant weight of shoot (SDW) and root (RDW) with the help of an electronic scale.

**TABLE 1:** Description of traits which collected manually and by DJ-GX02.

Trait name	Unit	Symbol	Measurement description
Shoot length	cm	SL	Manually (using a ruler)
Shoot dry weight	mg	SDW	Manually (using an electronic scale)
Root dry weight	mg	RDW	Manually (using an electronic scale)
Root dry weight per Shoot dry weight	mg	RDW/SDW	Manually (RDW divided by SDW)
Total plant biomass	mg	TPB	Manually (RDW plus SDW)
Total root length	cm	TRL	Measured electronically (DJ-GX02)
Surface area	cm^2^	SA	Measured electronically (DJ-GX02)
Average diameter	mm	AD	Measured electronically (DJ-GX02)
Root volume	cm^3^	RV	Measured electronically (DJ-GX02)
Branching number	–	BN	Measured electronically (DJ-GX02)

### Statistical Analysis

Statistical analysis of variance (ANOVA) with repeated values for all the above-mentioned traits at each stage in the natural population of spring soybean was carried out to assess the significance of variability amongst the soybean germplasm. The Pearson correlation coefficients among traits and across stages were analyzed to determine the relationship between seedling traits. Based on entry mean, the broad-sense heritability (H^2^) was estimated for each variable from variance estimates using the following equation (Pace et al., 2015):

$$H^{2}\frac{\delta_{G}^{2}}{\delta_{P}^{2}},\delta_{G}^{2}=\left(\frac{MSG-MSE}{rep}\right),\delta_{P}^{2}=\left(\frac{MSG-MSE}{rep}\right)+MSE$$

Where, $\delta_{G}^{2}$is genotypic variance $\delta_{P}^{2}$ is phenotypic variance, MSE is mean square error, MSG is mean square genotype, and rep (rep = 3) is the number of repetitions per accession. The Mean values of each line were used to examine the distribution of each trait across the three stages. All statistical analyses were performed using SPSS Statistics v.21 (IBM Corporation, MO, United States).

### Genotyping of Soybean Germplasms

The 260 Spring Soybean germplasm were sequenced using SLAF-seq technology (Sun et al., 2013; Han et al., 2016) to generate molecular markers throughout the whole genome. The sequencing data have been deposited in the sequence read archive^2^ under the accession number PRJNA681350.

#### DNA Extraction From Soybean

All samples were collected from plants growing in the experimental fields at Jilin Agricultural University, Changchun, China. Cetyltrimethylammonium bromide (CTAB) technique (slightly modified), as described by Murray and Thompson (1980) was used for DNA extraction from young and healthy leaves of each soybean accession. NanoDrop Spectrometer (ND-1000 Spectrophotometer, Thermo Scientific) was used to determine the DNA concentration and consistency of all samples. For SLAF sequencing, the quantified DNA was diluted to 100 ng⋅μl-1.

#### Enzyme Digestion, SLAF Library Preparation, and Sequencing

In order to acquire more than 309244 SLAF tags (defined as an enzyme fragment sequence of 364–464 bp) per genome, restriction enzyme combinations were tested and selected using *in silico* digestion prediction using the following criteria: To obtain more than 309244 SLAF tags per genome, combinations of restriction enzymes were examined and selected using the following criteria in the silico digestion prediction (1) The proportion restriction fragments in the repeat sequence was as low as possible; (2) The distribution of enzyme fragments in the genome was as uniform as possible; (3) The complementarity between the length of enzyme fragment and the specific reference genome; and (4) The number of SLAF tags met the expected number. Based on these criteria, RsaI and HaeIII (NEB, Ipswich, MA, United States) were selected as restriction enzymes. To obtain the SLAF tags, DNA from each soybean accession was digested, followed by fragment end reparation, dual-index paired-end adapter ligation, PCR amplification, and target fragment selection for SLAF library construction (Sun et al., 2013). To evaluate the accuracy of the restriction enzymes digestion experiment, rice Nipponbare (*Oryza sativa ssp. Japonica*) data genome (374.30 MB) (download address^3^) was used as a control. Subsequently, the SLAF-seq was performed with an Illumina HiseqTM 2500 (Illumina, Inc., San Diego, CA, United States) at the Biomarker Technologies Corporation.^4^ To obtain the reads for each sample, the raw SLAF-seq data was analyzed using Dual-Index software (Kozich et al., 2013). After filtering out the adapter reads, the sequencing accuracy was evaluated by measuring the guanine-cytosine (GC) content and Q30 (*Q* = −10 × log_10_(P)); suggesting a 0.1% risk of error and therefore 99.9% confidence). Ultimately, based on the sequence similarity, all SLAF paired-end sample reads were grouped via BLAT software (Kent, 2002). Polymorphic SLAF tags showed sequence polymorphisms between different accessions. High-consistent SLAF tags that show polymorphisms between accessions were then mapped to the soybean reference genome using BWA software (Li and Durbin, 2009).

#### Identification of SNPs

SNP loci were identified based on the polymorphic SLAF tags information using primarily GATK (McKenna et al., 2010). Based on clean reads mapped against the reference genome, local realignments were performed, and SNPs were identified using GATK software. For the detailed process, see GATK’s official website, https://www.broadinstitute.org/gatk/guide/best-practices.php. To ensure the reliability of SNPs identified using GATK, SAMtools also was used to detect SNPs with reference to Li et al. (2009). We designated the intersection of SNPs detected by both GATK and SAMtools as the reliable set of SNPs to subject to further analysis. Ultimately, highly consistent SNPs were selected with the criteria of minor allele frequency (MAF) > 0.05 and marker integrity frequency > 80% (Zhou et al., 2017) for genetic evolution correlation analysis, linkage disequilibrium analysis, and GWAS analysis.

### Population Structure and Linkage Disequilibrium Analysis

The population structure (*Q* matrix) was analyzed using Admixture software (Alexander et al., 2009). The number of subgroups (*k*-value) was set in from 1 to 15. The cluster results were cross-validated and the optimum number of subgroups was determined according to the valley value of the cross-validation error rate (Fu and Perry, 2020). A total of 179,960 SNP markers were used to assess the taxonomic and evolutionary relationships between the 260 genotypes via phylogenetic and principal component analyses. To perform the phylogenetic analysis, using the genomic data from the population, the distance between the materials was inferred based on the distance matrix, and then the phylogenetic tree was constructed. Based on SNPs, principal component analysis (PCA) was carried out in order to get the clustering of the different genotypes. The phylogenetic tree was generated using MEGA5 (Tamura et al., 2011) software based on the neighboring method (neighbor-joining), using the Kimura 2-parameter model with bootstrap repeated 1,000 times. PCA was carried out using EIGENSOFT (Price et al., 2006) software. Through Plink2 (Purcell et al., 2007) software, the average decay of LD measured in base pairs was calculated by plotting r^2^ onto difference between the genetic distance of two base pairs using an *r*^2^-value of 0.1 as a cut-off.

### Genome-Wide Association Studies

To find out the underlying SNPs or genes associated with root and shoot related traits in a diverse soybean panel, a genome-wide association study was conducted. The association analysis was performed based on the phenotypic dataset and the highly consistent population SNPs (179,960). Using the adjusted means for observations on each accession, we used five different statistical GWAS models, GLM, MLM, CMLM of TASSEL 5.0 software (Bradbury et al., 2007), FaST-LMM software (Lippert et al., 2011), and EMMAX software (Kang et al., 2010) to detect the SNPs associated with root and shoot complex traits as well as to analyze their stability across the different stages. Since the Bonferroni correction (BC) for multiple testing (Huang et al., 2010; Yang et al., 2014; Pace et al., 2015) was too conservative and only a few significant SNPs were detected, markers with adjusted −log_10_ (*P*) > 4 (control threshold) and −log_10_ (*P*) > 5 (suggestive threshold) were considered as significant for SNP-trait association. Considering the complexity of root traits, markers that passed the threshold score or above the threshold −log_10_(P) was held to be significantly associated with the target trait.

### Identification of Candidate Genes and Expression Analysis

The candidate region of candidate genes was defined by the average LD decay distance or the LD block. High priority candidate genes were chosen based on the criteria that they should have a minimum of 22% phenotypic contribution and located no more than 55-bp upstream and downstream distance from significant SNPs. For expression analysis, spring soybean lines with extreme phenotypic differences in root branching number were chosen. Thus, four lines with high branching number (Z166, Z201, Z203, and Z232) and four lines with low branching number (Z038, Z049, Z059, and Z063) (Supplementary File 5) were selected among the 260 spring soybean panel as samples for RNA-extraction. The total RNA was isolated from 100 mg root tissues following a Trizol method while the quantity and quality of RNA were examined using a NanoDrop Spectrometer (ND-1000 Spectrophotometer, Thermo Scientific). cDNAs were reverse transcribed from the total RNA using a cDNA kit (All-in-OneTm First-Strand cDNA Synthesis kit, GeneCopoeiaTm) following the manufacturer’s standard protocol. The two-step qRT-PCR Thermo cycling conditions were an initial denaturation at 95°C for 30 s, followed by 40 cycles of 95°C for 5 s, 58°C annealing for 30 s and 72°C extension for 15 s and an infinite hold at 10°C. ActinII was used as internal control gene and the relative expression level of the candidate genes was calculated using the comparative 2^−△△*ct*^ method (Livak and Schmittgen, 2001). All Primer sequences were designed using Primer Premier 5.0 software^5^ (Supplementary File 6).

## Results

### Statistical Analysis of Phenotypic Data

All accessions were examined for root and shoot related traits at 7, 14, and 21 germination days, corresponding to VC, V1, and V2, respectively (Supplementary File 3). Results of analysis of variance (ANOVA) showed substantial variability (*P* < 0.001) among varieties for all the traits evaluated at the three stages (Table 2). The descriptive statistics of all traits for the 260 spring soybean lines are listed in Table 2. High variability among the different traits investigated was observed at all stages. The coefficient of variation (CV) ranged from 7.38 to 36.61% for all seedling traits at all stages (Table 2). The highest variation was observed for the BN at VC stage, while the lowest variation was performed by AD at V1 stage. The outcome of skewness and kurtosis of all traits at all stages indicate that the distribution of frequencies tended to a normal curve according to the Shapiro–Wilk test (Supplementary File 4). The broad-sense heritability (H^2^) of root and shoot related traits was estimated, and the values ranged from 27% for AD to 86% for SL, indicating that the observed variation in root related traits is genetically controlled in greenhouse conditions. The Pearson correlation coefficients of all ten seedling root and shoot related traits were also analyzed at each stage, and most of the traits displayed significant positive correlations with similar tendencies at all stages (*P* < 0.05, *P* < 0.01, Table 3). As shown in Table 3, correlations among the following six seedling root and shoot related traits, TRL, RDW, SDW, TPB, RV, and SA were especially high and positive at all the three stages. The highest positive significant correlation was found between TPB and SDW (0.97) and the lowest one between TRL and RDW/SDW (0.12) (*P* < 0.01 or *P* < 0.05, Table 3). Interestingly, the TRL and RDW were highly correlated with root SA at all stages (0.66–0.92, 0.68–0.77, respectively). In addition, positive and significant correlations were found between TPB, RDW, and SDW (*r* > 0.80) as well as between RV and SA (*r* > 0.86) at all stages (Table 3).

**TABLE 2:** Descriptive statistical results of seedling root and shoot related traits at three stages.

Traits	Stage	Mean	± SD	Skewness	Kurtosis	Range	CV (%)	H^2^ (%)
SL	VC	13.87	2.48	−0.01	−0.57	12.00	17.88	80
	V1	19.65	2.47	0.16	0.09	14.84	12.57	85
	V2	25.48	2.85	0.01	−0.08	17.84	11.19	86
RDW	VC	0.03	0.01	0.08	−0.58	0.05	33.33	55
	V1	0.11	0.02	−0.40	−0.05	0.13	18.18	56
	V2	0.20	0.03	0.36	2.84	0.26	15.00	43
SDW	VC	0.15	0.03	0.23	−0.16	0.17	20.00	64
	V1	0.32	0.06	−0.48	0.42	0.39	18.75	56
	V2	0.51	0.07	0.08	2.27	0.57	13.73	52
RDW/	VC	0.23	0.06	0.33	0.46	0.40	26.00	37
SDW	V1	0.35	0.06	0.56	0.73	0.31	17.14	47
	V2	0.39	0.05	0.18	1.17	0.36	12.82	53
TPB	VC	0.18	0.04	0.21	−0.21	0.22	22.22	66
	V1	0.43	0.08	−0.53	0.51	0.52	18.60	57
	V2	0.71	0.09	0.05	2.96	0.81	12.68	48
TRL	VC	193.35	53.95	0.15	−0.53	248.81	27.90	56
	V1	405.34	68.29	−0.41	0.09	380.43	16.85	41
	V2	582.49	75.81	0.44	0.54	424.67	13.01	38
SA	VC	72.14	21.4	0.10	−0.57	103.09	29.66	68
	V1	157.67	27.23	−0.22	−0.08	147.82	17.27	42
	V2	241.06	32.72	−0.03	0.60	206.12	13.57	43
AD	VC	1.18	0.1	0.07	0.08	0.57	8.47	28
	V1	1.22	0.09	0.02	0.06	0.54	7.38	27
	V2	1.31	0.14	−0.09	0.60	1.02	10.69	50
RV	VC	2.14	0.69	0.22	−0.60	3.36	32.24	61
	V1	4.82	1.07	0.06	0.14	5.62	22.20	45
	V2	7.94	1.58	0.36	1.35	11.43	19.90	48
BN	VC	129.07	47.25	0.33	−0.36	223.00	36.61	63
	V1	243.99	61.69	0.40	0.51	408.33	25.28	63
	V2	430.29	81.49	0.68	1.70	543.00	18.94	61

*SL = shoot length; SDW = shoot dry weight; RDW = root dry weight; TRL = total root length; AD = average diameter; RDW/SDW = root dry weight per shoot dry weight; TPB = total plant biomass; RV = root volume; SA = surface area; BN = branching number; SD = Std. dev; CV = coefficient of variation; H^2^ = Broad-sense heritability.*

**TABLE 3:** Pearson correlation coefficients among all seedling root and shoot traits at three stages.

		SL	SDW	RDW	RDW/SDW	TPB	TRL	SA	AD	RV
**VC Stage**										
	SDW	0.67**								
	RDW	0.49**	0.65**							
	RDW/SDW	0.18**	0.10	0.77**						
	TPB	0.68**	0.97**	0.80**	0.31**					
	TRL	0.54**	0.57**	0.69**	0.48**	0.65**				
	SA	0.55**	0.62**	0.77**	0.54**	0.71**	0.92**			
	AD	−0.02	0.24**	0.32**	0.23**	0.28**	0.02	0.26**		
	RV	0.49**	0.63**	0.80**	0.56**	0.73**	0.83**	0.96**	0.47**	
	BN	0.35**	0.37**	0.51**	0.40**	0.44**	0.80**	0.77**	−0.05	0.67**
**V1 Stage**										
	SDW	0.55**								
	RDW	0.25**	0.74**							
	RDW/SDW	−0.39**	−0.35**	0.34**						
	TPB	0.50**	0.97**	0.85**	−0.17**					
	TRL	0.19**	0.53**	0.65**	0.14*	0.59**				
	SA	0.18**	0.58**	0.75**	0.20**	0.66**	0.84**			
	AD	0.01	0.23**	0.34**	0.146*	0.27**	−0.03	0.39**		
	RV	0.16**	0.53**	0.72**	0.23**	0.61**	0.64**	0.90**	0.63**	
	BN	0.23**	0.41**	0.45**	0.04	0.45**	0.61**	0.64**	0.13*	0.55**
**V2 Stage**										
	SDW	0.42**								
	RDW	0.006	0.60**							
	RDW/SDW	−0.43**	−0.35**	0.52**						
	TPB	0.32**	0.97**	0.79**	−0.10					
	TRL	0.07	0.21**	0.41**	0.24**	0.29**				
	SA	−0.04	0.39**	0.68**	0.38**	0.51**	0.66**			
	AD	−0.12	0.26**	0.42**	0.23**	0.34**	−0.31**	0.46**		
	RV	−0.08	0.38**	0.67**	0.39**	0.51**	0.25**	0.86**	0.81**	
	BN	−0.03	0.05	0.21**	0.20**	0.10	0.42**	0.53**	0.19**	0.44**

SL = shoot length; SDW = shoot dry weight; RDW = root dry weight; TRL = total root length; AD = average diameter; RDW/SDW = root dry weight per shoot dry weight; TPB = total plant biomass; RV = root volume; SA = surface area; BN = branching number, * significant at *P* < 0.05 and ** significant at *P* < 0.01.

### Genotyping Analysis

**FIGURE 1:**
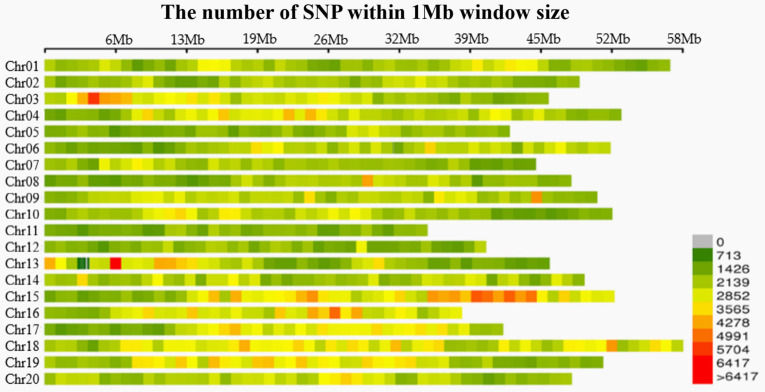
Distribution of SNPs in 20 chromosomes of Soybean. Each color represents different densities (Liu et al., 2020).

A total of 1263.91 Mb reads were obtained, and the cutting efficiency of RsaI-HaeIII (restriction enzyme for digestion) was 85.65%. The averages Q30 and GC content were 94.01 and 39.87%, respectively. The number of tags was recorded on the 20 soybean chromosomes (Supplementary File 8). Bioinformatics analyses revealed 1,102,987 SLAF tags, of which 609,361 polymorphic, and 2,311,337 SNP markers. Upon filtering the genotype results for minimum MAF of 0.05 and locus integrity of 0.8, a total of 179,960 highly consistent SNPs was obtained for GWAS, population structure and LD analyses, making the SNP density along the 20 chromosomes one SNP per 2.36 kb (Figure 1).

### Population Structure and Linkage Disequilibrium

**FIGURE 2:**
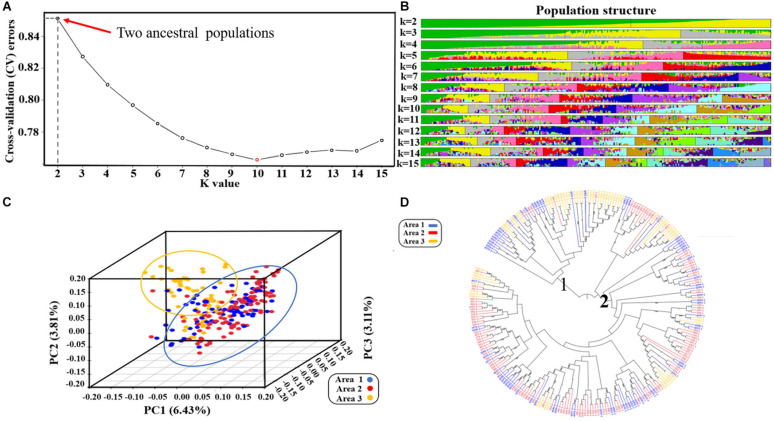
Population structure of 260 genotypes of spring soybean (Liu et al., 2020): **(A)** Diagram showing the value of 260 samples based on clustering from 1 to 15; Cross validation error rate for each *K*-value of 1–15, *X*-axis is *K*-value 1–15, *Y*-axis is cross-validation errors. **(B)** Clustering analysis when the number of subgroups is in the range 2–15, the colors represent separate groups. **(C)** Three-dimensional score plot (PCI, PC2, and PC3) to discriminate between soybeans lines from three provinces of China. **(D)** Phylogenetic tree of 260 soybean lines. Areal represents the soybean lines from Jilin province, Area2 represents the soybean lines from Heilongjiang province, Area3 represents the soybean lines from Liaoning province.

**FIGURE 3:**
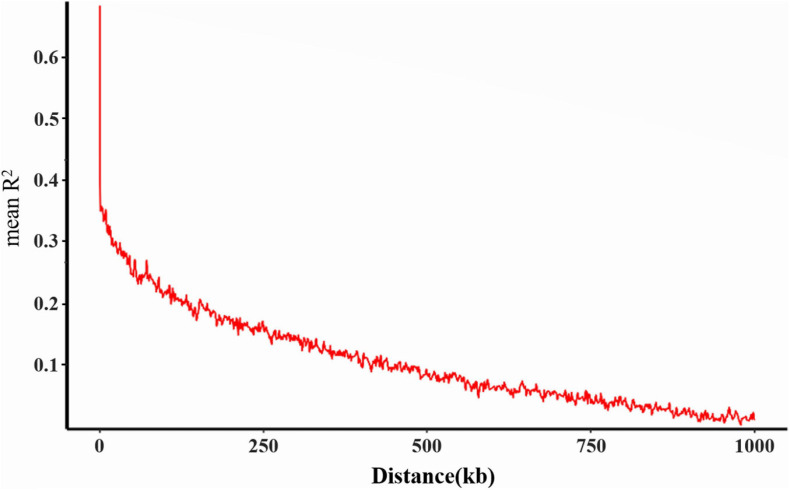
Genome-wide linkage disequilibrium (LD) decay for all 260 accessions. R^2^ indicates the squared allele frequency correlations between all pairs of SNP markers. The *X*-axis indicates the distance between marker pairs.

Based on the results of the cross-validation (CV) error rate and *K*-values from Admixture analysis, the 260 genotypes were subdivided into ten subgroups (*K* = 10, associated with the lowest CV error) originated from two ancestral lines (Figures 2A,B). These results have furthermore been validated by the phylogenetic tree and principal components analysis. From the phylogenetic tree, it can be concluded that the soybean lines originated from two large branches which suggests that the 260 soybean lines were from the same ancestors (Figure 2D). However, in the process of evolution, they evolved in different subgroups. By transforming a set of correlated variables into a set of linearly uncorrelated variables, PCA allows different subgroups to be clustered based on the degree of SNP difference of different materials. According to the PCA results, the 260 soybean lines can be divided into different subgroups with more overlapping areas. PC1, PC2, and PC3 accounted for 6.43, 3.81, and 3.11% respectively (Figure 2C). Collectively, it can be concluded that the 260 soybean lines were subdivided into 10 subgroups represented as an admixture of two ancestral populations. By spanning across all 20 chromosomes a subset of high-quality markers (see section “Materials and Methods”), we evaluated genome-wide LD in the 260 soybean accessions. The average decay distance of LD for all the 260 soybean lines was approximately 9.5 kb at a threshold of *r*^2^ = 0.3 (Figure 3).

### Genome-Wide Association Analysis

**FIGURE 4:**
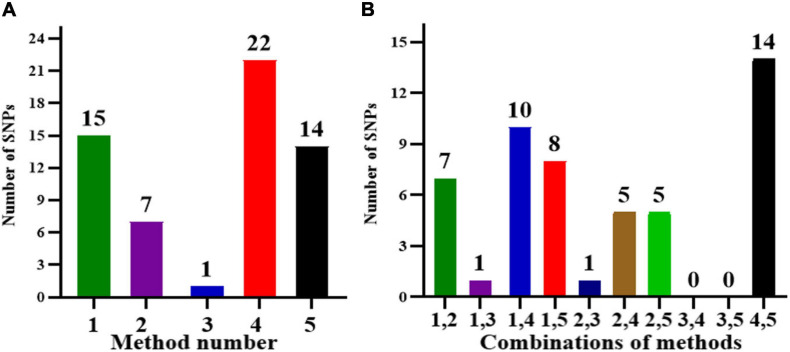
**(A)** The number of SNPs detected by different methods and **(B)** different combinations of methods. Method numbers correspond to (1) GLM, (2) MLM, (3) CMLM, (4) FaST-LMM, and (5) EMMAX.

In this analysis, as the multiple testing for BC was too conservative, to further confirm the significant SNPs associated with root related traits, all the 260 spring soybean genotypes were analyzed at three various stages using five GWAS models (GLM, MLM, CMLM, FaST-LMM, and EMMAX) in order to control false positives. According to the results of GWAS, a total of 27 unique SNP markers were detected which exhibited significant associations with root and shoot morphological traits at the critical threshold of −log_10_(*P*) ≥ 4.0, and these SNP positions were distributed in nine chromosomes, 2, 4, 6, 8, 9, 13, 16, 18, and 20 (Table 4). Minor allele frequencies (MAF) and phenotypic variation ranged from 5 to 47% and 20 to 72%, respectively, for all the identified SNPs (Table 4). We further analyzed significant SNPs that were repeatedly detected throughout the different stages and methods. In particular, 16 SNPs were consistently detected from two stages while 9 SNPs were detected by at least three GWAS models. These SNPs may therefore be considered major SNPs in this study. Comparing the results from the five GWAS approaches, 22 SNPs were detected by FaST-LMM, 15 SNPs by GLM, 14 SNPs by EMMAX, 7 SNPs by MLM, and 1 SNP by CMLM (Figure 4A and Table 4). The FaST-LMM detected the highest number of significant SNPs, with 22 SNPs detected for the ten traits, while CMLM was the least efficient by detecting only 1 SNP associated with RDW/SDW (Table 4). Besides, FaST-LMM combined with EMMAX followed by FaST-LMM combined with GLM were the combinations that detected the most number of SNPs with, 14 and 10 SNPs, respectively (Figure 4B). Interestingly, several SNPs were simultaneously detected by multiple GWAS models. The SNP with the lowest *p*-value, located on chromosome 16, position 29621947 (−log_10_(*P*) = 7.97, *R*^2^ = 72%), was associated with RDW/SDW and was detected across three different methods (GLM, MLM and CMLM) at VC stage. The second most important SNP found on Chromosome 13, location 40092616 (−log_10_(*P*) = 5.89, *R*^2^ = 34%) was associated with SDW and was detected across two different methods (GLM and MLM) at V2 stage. In regards of SNPs controlling multiple traits, two significant SNPs (Chr13-30887365, Chr09-40442312, −log_10_(*P*) = 4.64, 5.15, respectively) were found to be substantially associated with five specific seedling root and shoot traits namely AD, RDW, RV, TPB and SDW (Table 4). Individually, nine SNPs were significantly associated with SDW on chromosome 9 and 13, six SNPs were associated with SA on chromosome 4, 6, 13 and 20, four SNPs were associated with RV on chromosome 13 and 20, and three SNPs were associated with RDW on chromosome 9 and 14, three SNPs were associated with TPB on chromosome 8 and 13 (Table 4). The remaining five SNP positions were related to SL, BN and RDW/SDW along four different chromosomes 2, 9, 16, and 18 (Table 4). Additionally, Q-Q (quantile-quantile) plots and individual Manhattan plots of all the significant SNPs associated with all traits at all stages are shown in Supplementary Files 9, 10, respectively.

**TABLE 4:** Significant SNPs associated with root and shoot related traits detected by GWAS.

Trait	Position	Method^a^	Stage^b^	Chr	*P*-value	-log_10_(P)	R^2^	Allele	Count	MAF
TPB	4653989	1	1	Chr08	8.10E-05	4.09	0.251	T/C	T:116_C:22	0.16
TRL	4673257	4	1	Chr08	3.70E-05	4.43	0.252	C/G	C:123_G:21	0.15
BN	9585410	1	3	Chr18	1.22E-05	4.92	0.301	G/C	G:110_C:6	0.05
BN	11071047	5, 4	2	Chr02	6.17E-05	4.21	0.300	G/C	G:100_C:18	0.15
SA	12157467	4, 5	3	Chr06	2.18E-05	4.66	0.323	T/C	T:79_C:65	0.45
SA	12684509	4, 1, 5	2,3	Chr04	9.8E-05	4.01	0.238	A/T	A:93_T:45	0.33
RDW/SDW	29621947	2, 3, 1	1	Chr16	1.08E-08	7.97	0.721	C/A	C:113_A:15	0.12
SDW	30444845	2,1,4,5	2,3	Chr13	7.1E-05	4.15	0.260	T/C	T:130_C:8	0.06
SDW	30444857	2,1,4,5	2,3	Chr13	7.1E-05	4.15	0.260	G/A	G:130_A:8	0.06
SDW	30444862	5,1,2,4	2,3	Chr13	1.2E-05	4.92	0.240	A/G	A:130_G:8	0.06
SDW	30444865	4,1,2,5	2,3	Chr13	7.4E-06	5.13	0.354	C/T	C:130_T:8	0.06
SDW	30444924	1,2,4,5	2,3	Chr13	9.6E-05	4.02	0.207	C/T	C:130_T:8	0.06
RV, SDW, TPB	30887365	4, 5	2,3	Chr13	2.3E-05	4.64	0.234	A/T	A:123_T:11	0.08
RV	30910036	1	2,3	Chr13	6.1E-05	4.22	0.210	C/T	C:114_T:18	0.14
SA	30910036	5, 4	2,3	Chr13	5.2E-05	4.29	0.212	C/T	C:114_T:18	0.14
RV	30910036	4, 1, 5	2,3	Chr13	6.1E-05	4.07	0.253	C/T	C:114_T:18	0.14
SL	31398594	1, 4	2,3	Chr09	7.8E-05	4.11	0.228	T/C	T:118_C:8	0.06
TRL	33383414	4	2	Chr16	2.08E-05	4.68	0.213	G/T	G:76_T:68	0.47
RDW/SDW	36801463	5, 4	1	Chr16	1.26E-05	4.90	0.253	T/A	T:133_A:9	0.06
SDW	37605828	4	2	Chr09	9.04E-05	4.04	0.250	C/T	C:76_T:64	0.46
SDW	40092616	1, 2	3	Chr13	1.30E-06	5.89	0.345	C/G	C:105_G:31	0.23
AD, RDW, SDW, TPB	40442312	1, 4	1	Chr09	7.10E-06	5.15	0.306	C/A	C:131_A:7	0.05
RDW	40958088	1, 4, 5	2,3	Chr04	9.7E-05	4.01	0.264	A/G	A:117_G:7	0.06
SA	42374764	4	2,3	Chr20	6.1E-05	4.22	0.270	G/T	G:124_T:8	0.06
SA	42374776	4	2,3	Chr20	7.2E-05	4.14	0.250	A/T	A:124_T:8	0.06
SA	42374808	4	2,3	Chr20	7.2E-05	4.14	0.250	T/C	T:124_C:8	0.06
RV	45819633	4, 5	2,3	Chr20	7E-05	4.16	0.251	A/G	A:105_G:23	0.18

*SL = shoot length, SDW = shoot dry weight, RDW = root dry weight, TRL = total root length, AD = average diameter, RDW/SDW = root dry weight per shoot dry weight, TPB = total plant biomass, RV = root volume, SA = surface area, BN = branching number, ^*a*^ method 1–5 refers to GLM, MLM, CMLM, FaST-LMM, and EMMAX, respectively, ^*b*^ stage 1–3 refers to VC, V1, and V2, respectively, MAF = minor allele frequency, R^2^ = phenotypic contribution, Chr = chromosome.*

### Mining of Candidate Genes and Expression Analysis

**FIGURE 5:**
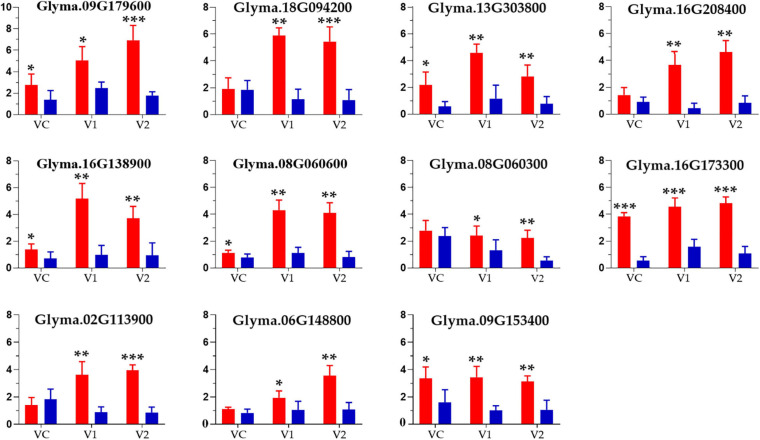
Relative expression levels of eleven candidate genes in phenotypically different root samples from the spring soybean at three seedling stages analyzed via qRT-PCR. The red and blue bars indicate the mean expression level (for four samples) of genes in high and low branching number accessions at VC, Vl and V2 stages, respectively; ***, **, and * specify the significance at the level of *P* < 0.001, *P* < 0.01, and *P* < 0.05, respectively.

In this analysis, eleven candidate genes located within ± 55 bp distance from the SNPs with phenotypic contribution rate (*R*^2^ > 22%) were obtained as putative candidate genes for root and shoot development in the spring soybean panel. The candidate genes were Glyma.09G179600, Glyma.08G060300, Glyma.16G138900, Glyma.16G208400, Glyma.09G153400, Glyma.13G303800, Glyma.08G060600, Glyma.16G173300, Glyma.02G113900, Glyma.06G148800, and Glyma.18G094200 (Table 5). The list of all genes associated with significant SNPs detected in this study is presented in Supplementary File 7. The functional annotations of these candidate genes were clarified and predicted using NCBI and SoyBase databases (Table 5). The relative expression level results obtained through qRT-PCR revealed that the eleven candidate genes were upregulated at all considered stages (VC, V1, V2) in the different root samples. Comparatively, expression levels in accessions with higher branching number were higher, especially in the later growth stages (Figure 5), indicating that these genes play crucial roles in soybean root development.

**TABLE 5:** Potential candidate genes identified through GWAS and their functional annotations.

Genes	Traits	SNP position	Length (bp)	Chr	Distance(bp)	Function explanation
Glyma.09G179600	AD, RDW, SDW, TPB	40442312	2525	Chr09	−55	PREDICTED: *Glycine max* aminopeptidase M1-like (LOC100811437), transcript variant X3, mRNA
Glyma.08G060300	TPB	4653989	2338	Chr08	−44	PREDICTED: *Glycine max* uncharacterized LOC100803004 (LOC100803004), misc_RNA
Glyma.16G138900	RDW/SDW	29621947	1464	Chr16	−48	PREDICTED: *Glycine max* sugar transporter ERD6-like 6-like (LOC100801076), mRNA
Glyma.16G208400	RDW/SDW	36801463	11522	Chr16	8	PREDICTED: *Glycine max* chloride channel protein CLC-c-like (LOC100809070), transcript variant X8, mRNA
Glyma.09G153400	SL	37605828	3135	Chr09	−41	Phaseolus vulgaris hypothetical protein (PHAVU_009G205900g) mRNA, complete cds
Glyma.13G303800	SDW	40092616	5464	Chr13	11	PREDICTED: *Glycine max* G-type lectin S-receptor-like serine/threonine-protein kinase At4g03230-like (LOC100817898), mRNA
Glyma.08G060600	TRL	4673257	3725	Chr08	24	PREDICTED: *Glycine max* pentatricopeptide repeat-containing protein At2g17140-like (LOC100803535), transcript variant X2, misc_RNA
Glyma.16G173300	TRL	33383414	832	Chr16	−49	*Glycine max* strain Williams 82 clone GM_WBb0117F02, complete sequence
Glyma.02G113900	BN	11071047	3810	Chr02	1	PREDICTED: *Glycine max* tankyrase-2-like (LOC100795610), transcript variant X1, mRNA
Glyma.06G148800	SA	12157467	3573	Chr06	−17	PREDICTED: *Glycine max* ribonuclease P protein subunit p25-like protein-like (LOC100802967), transcript variant X1, mRNA
Glyma.18G094200	BN	9585410	2581	Chr18	19	PREDICTED: *Glycine max* disease resistance protein RPM1-like (LOC102663437), mRNA

## Discussion

Our study’s main goal was to elucidate the genetic basis underlying the phenotypic differences in spring soybean root system architecture. Our study described a genome-wide association mapping for root and shoot related traits in a diverse population consisting of 260 natural soybean genotypes. China has preserved 70% of its unique soybean germplasm in the global gene bank, and many of them are ancient local varieties with high genetic diversity (Qiu et al., 2011). The genetic diversity and widespread phenotypic heterogeneity in such germplasms improve the reliability of GWAS for discovering significant SNPs underlying root traits. Although the optimization of crop root systems has been projected for a long time, genetic dissection and improvement of roots are seldom attempted (Bishopp and Lynch, 2015). Phenotyping of root architecture traits at the adult stage in the field is a complex, costly, and time-consuming task, particularly when a large number of plants need to be phenotyped (Huisman, 1982). Root evaluation methods based on controlled environments such as greenhouse are less time-consuming, and reliable results easily related to the field can be achieved (Huisman, 1982). Thus, 260 soybean inbred lines were grown under standardized conditions in the greenhouse with three replications in order to get accurate and reliable phenotypes at different seedling stages. The experiment was conducted in three stages (VC, V1, V2) which corresponded to 7, 14, and 21 days after germination (Table 2).

Large phenotypic variations for the ten vegetative period traits can be observed in the 260 spring soybean accessions (Table 2). Our research showed that the phenotypic output of all the investigated root related traits rapidly increased from stage VC to stage V2, supporting the crucial role of the selected stages in root and shoot evaluation in this study. The broad-sense heritability of most root related traits was moderate to high (Table 2). In addition, the majority of traits followed a normal distribution indicating the relative stability behind the measured features in this study. Furthermore, all the root related traits were highly significantly and positively correlated at all studied stages. Interestingly, root dry weight (RDW) and total root length (TRL) were extremely correlated with the surface area of root at all stages, which affects the nutrient of plant and water absorption (Comas et al., 2013). In this study, total root length and volume were found tightly correlated. The involvement of total root length and volume in soybean has so far been reported to be associated with aluminum tolerance and drought (Liu et al., 2005b; Yang et al., 2005; Ying et al., 2007). Acquisition of nutrients from soil and accessibility of soil moisture by plants was stated to be more reliant on root length and/or surface area than total root biomass (Nye and Tinker, 1977; Sattelmacher et al., 1990). Also, these traits were previously indicated to regulate the total root growth rate and the root architecture plasticity of plants, which is necessary for successful soil exploration to intercept nutrients and communicate stress signals (Falik et al., 2012). Several studies in crops have emphasized that a deeper root system is influenced by root dry weight and total root length, important for improving drought tolerance and being associated with the final yield under drought stress in soybean (Hudak and Patterson, 1995) and rice (Bengough et al., 2011; Suji et al., 2012). Therefore, identifying ideotypes for root traits involved in drought tolerance at early growth stages could be very useful in guiding the development of soybean cultivars with enhanced soil exploration, and thus water acquisition, under water deficit conditions (Dayoub et al., 2021). In this study, four soybean genotypes including Z166, Z201, Z203 and Z232 with promising root structure were identified. The different genotypes performed better in all the key root architectural traits investigated like, total root length, root branching number, root surface area, root volume, and root dry weight (Supplementary File 5). These accessions are all Chinese landraces, with Z166 from Jiangsu Province and Z201, Z203, and Z232 from Jilin Province (Supplementary File 1).

Understanding the mechanism of root related traits in crops like soybean through SNPs and genes investigation could be the most powerful and successful strategies to develop high-quality root cultivars via marker-assisted selection. Many studies have been performed to elucidate the genetic foundation and underlying root genetic function in soybean, and several root-related QTLs have been reported in various populations using various environments (Liang et al., 2010, 2014; Lü et al., 2010; Rong et al., 2011). Nevertheless, most of these QTL mapping studies were carried out using low-density genetic linkage maps (Karikari et al., 2019). In soybean, roots have previously been indicated to be controlled by multigenes, and are strongly affected by environmental changes (Lü et al., 2010). Hence, most of the earlier recorded QTLs were neither stable nor confirmed and have therefore not been successfully utilized in marker-assisted selection (MAS) for soybean breeding. To address these limitations, a genome-wide association study based on LD was performed in the present study. Previous studies have shown that GWAS is an effective approach to detecting and identifying SNPs or genes correlated with specific traits, such as root growth in crops (Atwell et al., 2010; Ingvarsson and Street, 2011; Riedelsheimer et al., 2012; Crowell et al., 2016). Based on 179,960 high-quality SNPs in 260 spring soybean accessions, 27 SNPs were identified to be associated with ten root related traits with the significance threshold of (−log_10_(*P*) ≥ 4). Many SNPs found in this analysis were not recorded in earlier studies. This might be clarified by the variance in the genetic background as well as the environmental factors that influence root related traits expression and, ultimately, plant development.

In the present research, besides the most commonly used models, three GWAS models including CMLM, FaST-LMM, and EMMAX were used to improve the accuracy of the results. As GLM uses only Q population structure information while MLM uses Q + K, due to the strict screening requirements, CMLM and MLM may increase GWAS analysis accuracy. Many researchers have reported that GLM’s stringency and accuracy are weaker compared to the MLM (Huang et al., 2010; Yang et al., 2010; Zhang et al., 2010; Liu et al., 2016). Furthermore, it had been previously recommended that multiple algorithmic models ought to be used to perform GWAS analysis in real application for complex traits studies (Dhanapal and Crisosto, 2013; Hecht et al., 2013; Zhang H. et al., 2017) due to the limitation in some GWAS scanning models to detect associations with the variation of polygenes. The EMMAX model (Kang et al., 2010) has the ability to handle a large number of markers and reduces the computational time for analysis; FaST-LMM (Lippert et al., 2011) can solve the computational problem but requires that the number of individuals be more than the number of SNPs; and CMLM (Zhang et al., 2010) can boost both statistical power and computing efficiency (speed). Based on these GWAS methods, a different number of SNPs were detected as each method has its specific power and stringency to identify SNP associations. In this study, FaST-LMM followed by GLM were the most effective in detecting more significant SNPs associated with the ten target traits as compared to other models. Thus, 22 SNPs were detected by FaST-LMM, 15 by GLM, 14 by EMMAX, 7 by MLM and 1 by CMLM (Figure 4A and Table 4), and among the combinations of methods, FaST-LMM combined with EMMAX detected the maximum number of SNPs (Figure 4B). Comparatively FaST-LMM detected the greatest number of SNPs followed by GLM, and EMMAX while the small number of SNPs was detected by CMLM and MLM models which confirmed the relevance and ability of the different GWAS models used for controlling the false positives. Several studies used various approaches, along with significant and suggestive level to balance the false negatives and false positives in root related studies (Li X. et al., 2017; Jia et al., 2019). Interestingly, a major SNP, Chr09-40442312 was found to be associated with four root-related traits AD, RDW, SDW and TPB, and described 30.6% of the total phenotypic variation. The above-mentioned major SNP overlapped one annotated gene Glyma.09G179600, which encodes an amino acid transport and metabolism. This gene was also previously recorded for soybean nodule-related traits (Li et al., 2018). Thus, this gene may be a promising candidate gene for further study on soybean root development.

Chromosome 13 was found substantially associated with numerous SNPs for multiple root and shoot related traits including RDW, SDW, RV, TPB, and SA. Of these SNPs, Chr13-30444845, Chr13-30444857, Chr13-30444862, Chr13-30444865, Chr13-30444924, Chr13-30887365, and Chr13-30910036 overlapped with the reported QTLs ‘Root length 1-1′, ‘Root length 1-2′, ‘Root length 1-3′, ‘Root length 1-4′, ‘Root area 2-1′, and ‘Root area 2-2′ underlying soybean root length and surface area development under hypoxia conditions (Van Nguyen et al., 2017). The soybean salt tolerance loci ss715616115 and ss715624611 on chromosomes 13 (position 33,415,484) and 16 (position 33383414) reported by Zeng et al. (2017) were located especially near the SNPs Ch13-40092616 and Chr16-33383414 associated with SDW and TRL, respectively. SNP Chr13-40092616 was only 11 bp far from the gene model Glyma.13G303800 coding for a G-type lectin S-receptor-like serine/threonine-protein kinase At4g03230-like. This gene was earlier indicated to show high expression in soybean seedling leaves (Libault et al., 2010; Severin et al., 2010). Chromosome 8 harbored SNPs Chr8-4673257 and Chr8-4653989 for two different traits namely TRL and TPB, respectively. SNP Chr8-4673257 was located 24 bp from the candidate gene Glyma.08G060600 predicted to encode a pentatricopeptide repeat-containing protein At2g17140-like highly expressed in nodule roots, leaves and primary root in soybean (Libault et al., 2010; Severin et al., 2010). SNP Chr8-4653989 was located 44 bp downstream the gene model Glyma.08G060300 highly expressed in the soybean primary root and lateral roots (Libault et al., 2010; Severin et al., 2010). Furthermore, several QTLs for root dry weight, taproot length, shoot length, lateral root number, and total root length were previously detected on this linkage group from various similar root studies at seedling stage/time-point (Liu et al., 2005a; Ying et al., 2007; Liang et al., 2014; Manavalan et al., 2015; Prince et al., 2020). Moreover, a region spanning only 15 cM on this chromosome was earlier revealed to contain four cluster QTLs for root fresh weight, root dry weight, shoot fresh weight, and shoot dry weight using the Essex × Forrest soybean population (Brensha et al., 2012). SNPs Chr6-12157467, Chr18-9585410, and Chr20-45819633 associated with SA, BN, and RV were in LD with three noteworthy QTNs namely q6, q18-2, and q20-3 associated with photosynthetic traits related to phosphorus efficiency in soybean, respectively (Lü et al., 2018). SNP Chr6-12157467 was located 17 bp downstream the gene model Glyma.06G148800 predicted to encode a ribonuclease subunit p25-like protein-like while SNP Chr18-9585410 was 19 bp away from the candidate gene Glyma.18G094200 encoding for a RPM1 protein-like conferring disease resistance. These two genes were recorded to display high expression in soybean primary root (Libault et al., 2010; Severin et al., 2010). Chromosomes 6 and 4 seem to harbor multiple QTLs for soybean root traits. Wang et al. (2004) detected 3 QTLs for root weight on chromosomes 6 with 6.8–26.3% of the phenotypic contributions at maturity stage of soybean. Liu et al. (2005b) revealed 6 QTLs for drought tolerance on chromosome 6 at V4 stage, including 3 major QTLs explained 22 to 24.7% of phenotypic variations associated with root dry weight, total root length, and root volume. At R7 stage, Zhou et al. (2009) mapped 1 and 3 QTLs for root weight on chromosomes 6 and 8, respectively, with phenotypic contributions ranged from 8.3 to 12.5%. Our detected SNP Chr06-12157467 associated with SA at V2 stage on chromosome 6 was also found very close to *qMRL6*, a QTL for main root length linked to the marker locus Sat_153 (10.52 cM), which was earlier detected in a similar study at V2 stage (Rong et al., 2011). In addition, this SNP was in LD with *qMRL/RW6*, a QTL for main root length to root weight ratio (MRL/RW) linked to marker Satt557 (5.55 cM) (Rong et al., 2011). SNP Chr04-40958088 on chromosome 4 associated with RDW and consistently detected at V1 and V2 stages collocated with *qHW4* (Rong et al., 2011), a previously reported QTL for hypocotyl weight between markers Satt476 and Satt294. This SNP was also in LD with a SNP on chromosome 4 (position 36376946) associated with root surface area detected by Prince et al. (2019) at V1 stage. SNPs Chr16-29621947 and Chr16-33383414 on chromosome 16 significantly associated with RDW/SDW and TRL, respectively were in LD with a SNP (position 30021671) for lateral root number and root diameter in thickness Class I (Prince et al., 2019). SNPs chr20-42374764, chr20-42374776, chr20-42374808 associated with SA and SNP chr20-45819633 associated with RV were found very close to the SNP (position 43048714) associated with root diameter in volume and surface area Class II (Prince et al., 2019). These loci were also in LD with a QTL for total root length recently identified by Prince et al. (2020) within a SNP06258 ∼ NCSB_004833 marker interval.

Based on the LD decay analysis, an approximately 55 bp SNP region was chosen as a candidate region for selecting potential candidate genes. According to the functional annotation, GO enrichment, and gene expression profile, we selected 11 genes for expression analysis using qRT-PCR, widely used to validate GWAS-identified genes expression with high accuracy and sensitivity (Bustin, 2002; Bustin and Nolan, 2004). The relative expression rates of the eleven potential candidate genes tested were significantly higher in high root branching number accessions as compared to low root branching number accessions at all considered growth stages (VC, V1, V2), particularly later stages (Figure 5). Therefore, the evaluated genes served as a constructive regulator in the spring soybean panel which could be of great significance for elite’s soybean breeding programs. Upcoming research based on these potential candidate genes will, however thoroughly elucidate the role of these aforementioned genes in soybean root growth and development.

## Conclusion

In conclusion, this work provides a comprehensive analysis of the genetic architecture of root related traits in spring soybean. We performed a genome-wide association study through 260 soybean accessions genotyped by SLAF-seq for root related traits at three developmental stages under standard greenhouse conditions and identified a total of 27 substantial SNPs using different GWAS models (GLM, MLM, CMLM, FaST-LMM, and EMMAX). All The detected SNPs showed individual phenotypic contribution rates greater than 20% and were significantly associated with SDW (7), SA (6), RV (3), TRL (2), BN (2), RDW/SDW (2), TPB (1), RDW (1), and SL (1). However, two major SNPs were found to be associated with multiple traits including AD, RDW, SDW, and TPB in one side and RV, SDW, TPB in the other. Based on the LD region of no more than 55-bp and SNPs with phenotypic contribution rate greater than 22%, eleven promising root and shoot regulating genes viz. Glyma.09G179600, Glyma.08G060300, Glyma.16G138900, Glyma.16G208400, Glyma.09G153400, Glyma.13G303800, Glyma.08G060600, Glyma.16G173300, Glyma.02G113900, Glyma.06G148800, and Glyma.18G094200 overlapping with SNPs on chromosomes 2, 6, 8, 9, 13, 16, and 18 were detected and verified for expression level. The evaluated genes were shown to serve as positive regulators for root branching number in the spring soybean panel, which could play a crucial role in marker-assisted selection programs. Thus, SNPs and underlying genes discovered in the current study provide a key basis for revealing the complex genetic mechanism of root development and genetic enhancement of soybean root, as well as supporting the breeding of high-efficiency soybean root system varieties in the future.

## Data Availability Statement

The original contributions presented in the study are publicly available. This data can be found here: https://www.ncbi.nlm.nih.gov/bioproject/PRJNA681350.

## Author Contributions

AM drafted the original manuscript. AAM reviewed and edited the manuscript. AM and AAM conducted the experiments and performed the analysis. JQ, QZ, and YD performed the phenotyping. GA and NA conducted formal analysis. PW supervised the experiments. All authors have read and agreed the final version of the manuscript.

## Conflict of Interest

The authors declare that the research was conducted in the absence of any commercial or financial relationships that could be construed as a potential conflict of interest.

## Publisher’s Note

All claims expressed in this article are solely those of the authors and do not necessarily represent those of their affiliated organizations, or those of the publisher, the editors and the reviewers. Any product that may be evaluated in this article, or claim that may be made by its manufacturer, is not guaranteed or endorsed by the publisher.

## Footnotes

1
www.Dianjiangtech.com

2
https://www.ncbi.nlm.nih.gov/sra

3
http://rapd.dna.affrc.go.jp/

4
http://Biomarker.com.cn/

5
http://www.premierbiosoft.com

## Supplementary Material

Supplementary File 1 List and provenance of the 260 spring soybean accessions.

Supplementary File 2 Root trait phenotypic evaluation and scanning.

Supplementary File 3 Phenotypic data of root and shoot related traits of 260 spring soybean population at VC, V1, and V2 stages.

Supplementary File 4 Histogram of the frequency distribution of root and shoot related traits at all three stages with data from VC stage shown in blue color **(A)**, data from V1 stage shown in green color **(B)**, and data from V2 stage shown in turquoise color **(C)**.

Supplementary File 5 Accessions used in qRT-PCR analysis.

Supplementary File 6 List of primers used for the qRT-PCR assay of the investigated genes involved in the root pathway.

Supplementary File 7 List of all associated genes with significant SNPs detected in this study.

Supplementary File 8 Distribution of SLAF tags and polymorphic SLAF tags on soybean chromosomes.

Supplementary File 9 Quantile-Quantile (Q-Q) plots of root and shoot related traits at three stages.

Supplementary File 10 Individual Manhattan plots of all the significant SNPs associated with all traits at all stages.
