# Supplementary material for: Genome-Wide Association Study of Root and Shoot Related Traits in Spring Soybean (Glycine max L.) at Seedling Stages Using SLAF-Seq
Source: Front Plant Sci. 2021 Jul 28;12:568995. doi: 10.3389/fpls.2021.568995 (PMC8355526; doi:10.3389/fpls.2021.568995)
Supplement: Supplementary File 1 — List and provenance of the 260 spring soybean accessions. [file Data_Sheet_1.ZIP › Supplementary File 6.docx]

**Table S6.**

| **Name of Genes** | **Primer Sequences** | **Use** |
| --- | --- | --- |
| Glyma.09G179600 S  Glyma.09G179600 AS | GCTGCTTGCAAGGCTACTTT  GTGCAACCCATGGATCCTCA | qRT-PCR assay |
| Glyma.08G060300 S  Glyma.08G060300 AS | CGGTTCCTATGCAAACCCCT  ACGCCCGCTCCATATACTTG | qRT-PCR assay |
| Glyma.16G138900 S  Glyma.16G138900 AS | AGGAGCAACAGTCAGTGGTC  TAGAAATGGCGAGCCATCCG | qRT-PCR assay |
| Glyma.16G208400 S  Glyma.16G208400 AS | GGCTCCAAGCACCCTTTTTG  GCCGGTCTCGGTCATTTTTG | qRT-PCR assay |
| Glyma.09G153400 S  Glyma.09G153400 AS | GGAAGCTGGTTGAGACCCTC  CTCATCAATTGCACGGGCTG | qRT-PCR assay |
| Glyma.13G303800 S  Glyma.13G303800 AS | CTTGGCTACCAGCACCAGAA  GTTTGAGTCACAACGGCACC | qRT-PCR assay |
| Glyma.08G060600 S  Glyma.08G060600 AS | CGGTTTCGTCTCGTGGCTAT  ACGAGGATCCCGAGAGTGAA | qRT-PCR assay |
| Glyma.16G173300 S  Glyma.16G173300 AS | TGTTAGTTGTCCCTGGTTACTCA  CAGCAAAACTGGCAAGGAGAA | qRT-PCR assay |
| Glyma.02G113900 S  Glyma.02G113900 AS | TCAAAGCTGCAGAAGAGGGC  GACGTGGAGAAGGGAACGAG | qRT-PCR assay |
| Glyma.06G148800 S  Glyma.06G148800 AS | TGATGATTGGGATGGTGGGC  TAGTACCCAACTGGCTGCAC | qRT-PCR assay |
| Glyma.18G094200 S  Glyma.18G094200 AS | AATAGGCGCATTGCACAACC  CTCCATTTGAACCCGACCCA | qRT-PCR assay |
| ActinII1S  ActinII1AS | GCTGTTCTTTCACTTTATGCAAG  CGCTCGGCTGAGGTGGTGAAGGA | Reference gene |

List of primers used for the qRT-PCR assay of the key structural genes involved in root related traits.
