# Supplementary material for: Genome-Wide Association Study of Root and Shoot Related Traits in Spring Soybean (Glycine max L.) at Seedling Stages Using SLAF-Seq
Source: Front Plant Sci. 2021 Jul 28;12:568995. doi: 10.3389/fpls.2021.568995 (PMC8355526; doi:10.3389/fpls.2021.568995)
Supplement: Supplementary File 1 — List and provenance of the 260 spring soybean accessions. [file Data_Sheet_1.ZIP › Supplementary File 8.docx]

**Table S8.** Distribution of SLAF tags and polymorphism SLAF tags on soybean chromosomes.

| **Chromosome ID** | **SLAF number** | **Polymorphic SLAF** |
| --- | --- | --- |
| **Chr01** | 68,053 | 35,562 |
| **Chr02** | 54,347 | 29,044 |
| **Chr03** | 51,354 | 30,338 |
| **Chr04** | 61,910 | 35,028 |
| **Chr05** | 48,107 | 23,810 |
| **Chr06** | 57,097 | 31,523 |
| **Chr07** | 49,885 | 26,528 |
| **Chr08** | 52,881 | 27,572 |
| **Chr09** | 57,347 | 31,924 |
| **Chr10** | 59,171 | 32,718 |
| **Chr11** | 38,028 | 18,205 |
| **Chr12** | 44,930 | 22,111 |
| **Chr13** | 50,187 | 27,274 |
| **Chr14** | 57,351 | 30,853 |
| **Chr15** | 56,928 | 35,099 |
| **Chr16** | 42,395 | 25,701 |
| **Chr17** | 46,492 | 27,743 |
| **Chr18** | 65,252 | 39,305 |
| **Chr19** | 58,826 | 34,837 |
| **Chr20** | 54,917 | 30,012 |


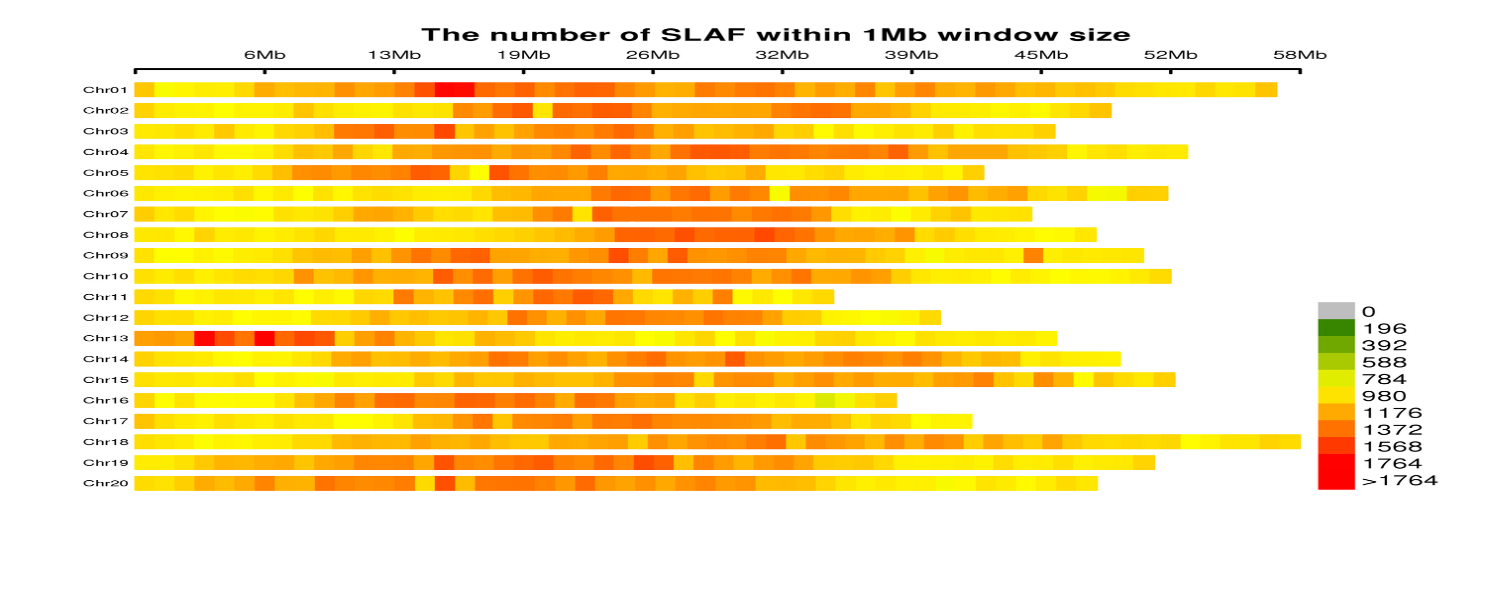


**Figure S8.** Distribution map of SLAF on chromosomes, each color represents different densities.
