# Supplementary figures and images for: Genome-Wide Association Study of Root and Shoot Related Traits in Spring Soybean (Glycine max L.) at Seedling Stages Using SLAF-Seq
Source: Front Plant Sci. 2021 Jul 28;12:568995. doi: 10.3389/fpls.2021.568995 (PMC8355526; doi:10.3389/fpls.2021.568995)

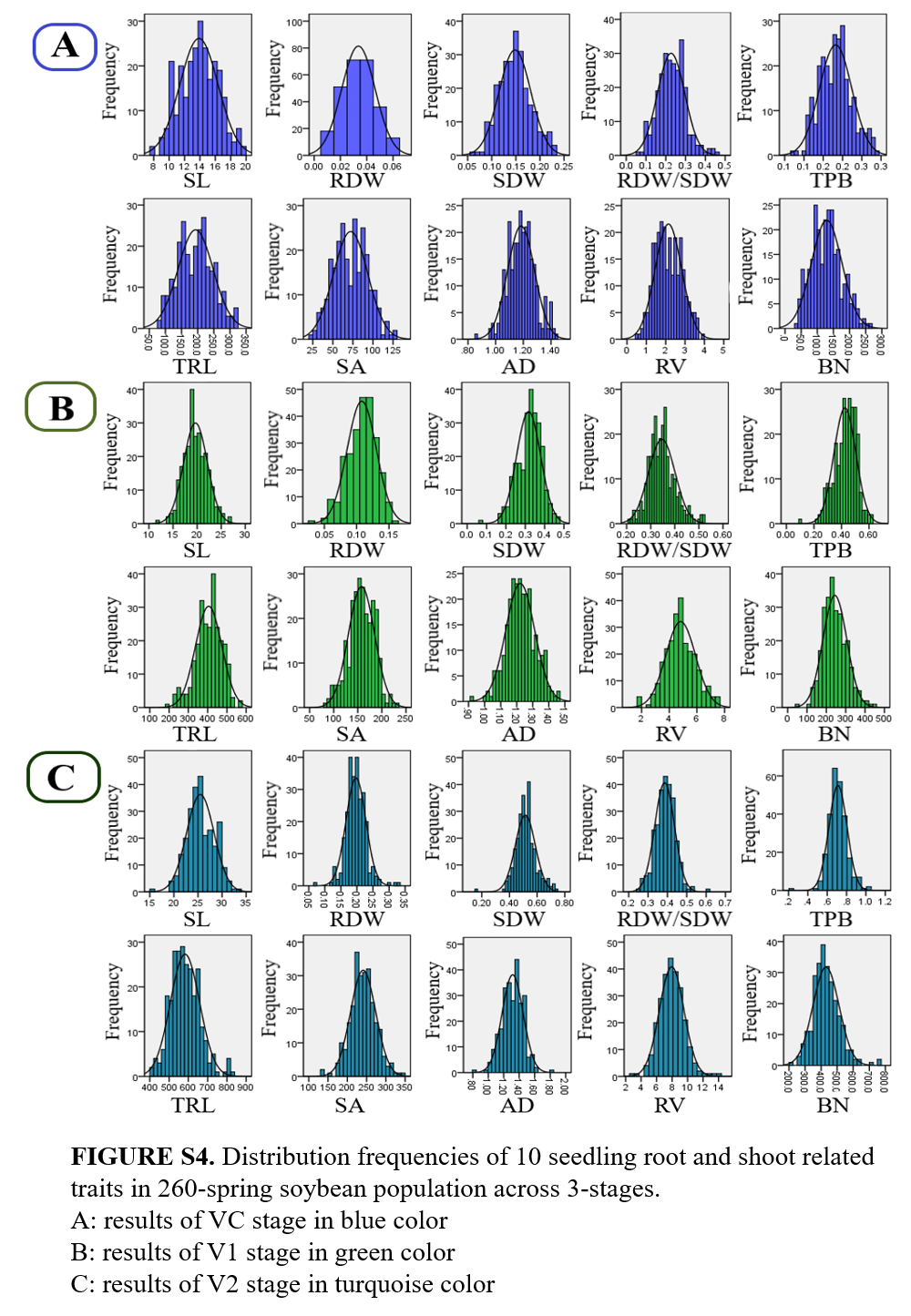

Supplement: Supplementary File 1 — List and provenance of the 260 spring soybean accessions. [file Data_Sheet_1.ZIP › Supplementary File 4.docx]

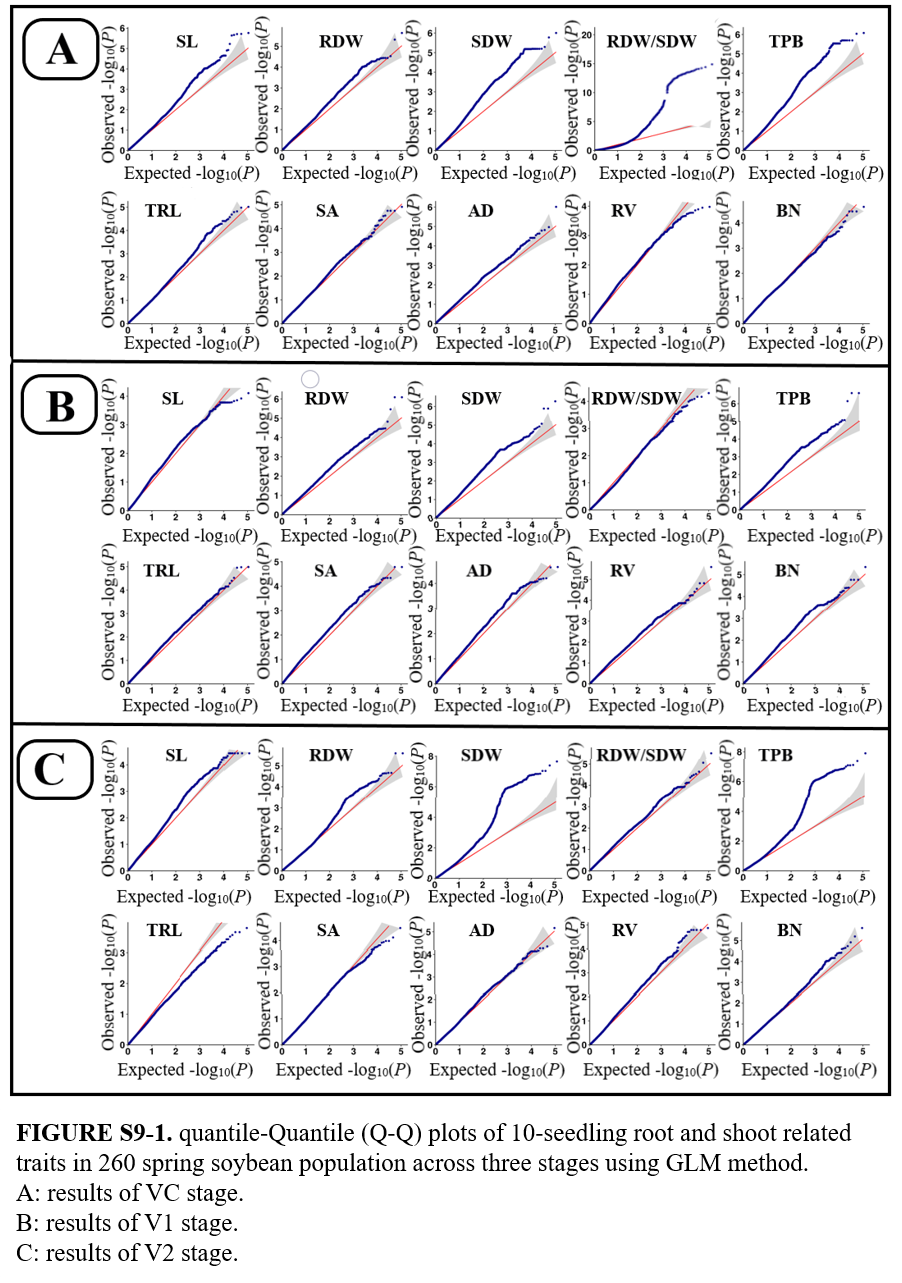

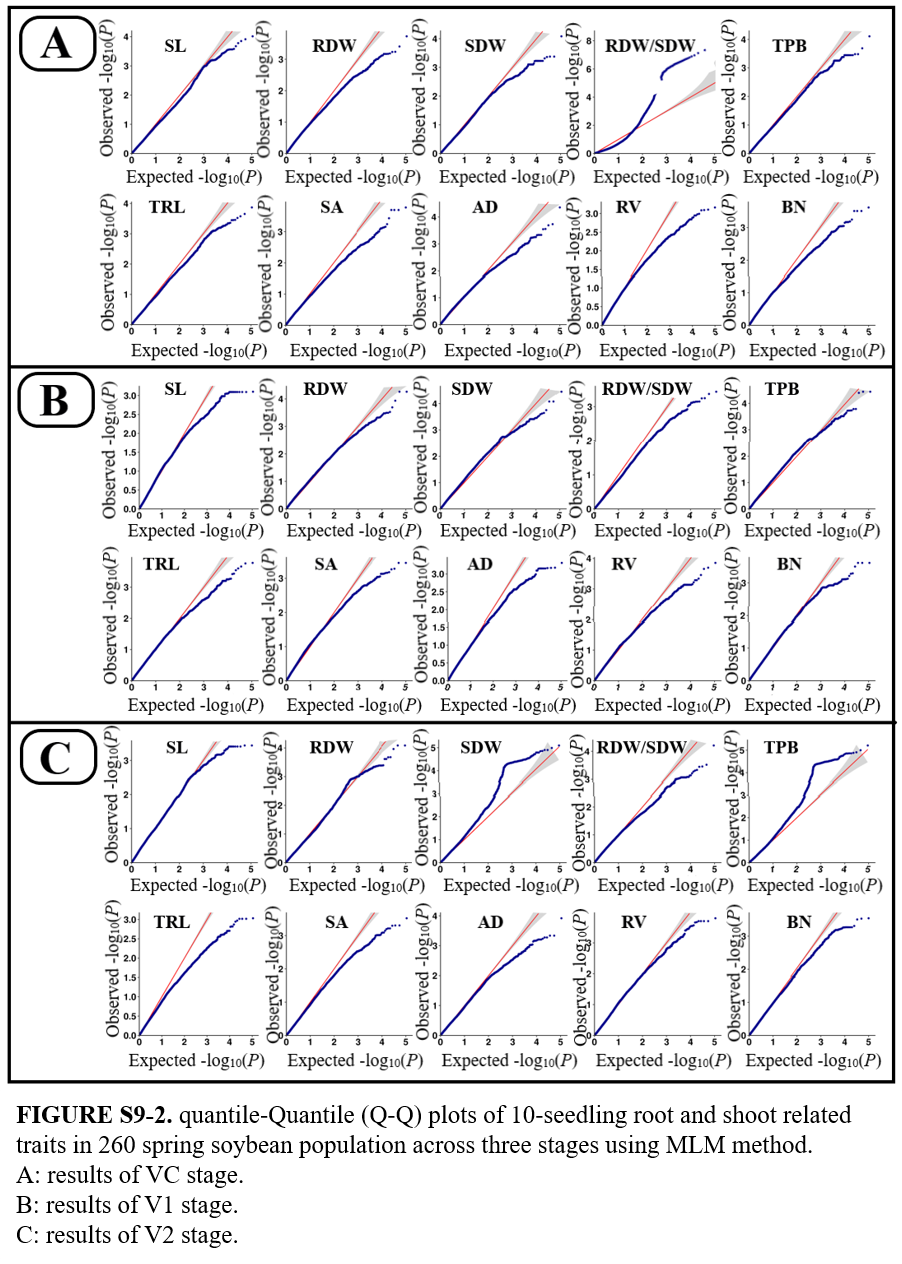

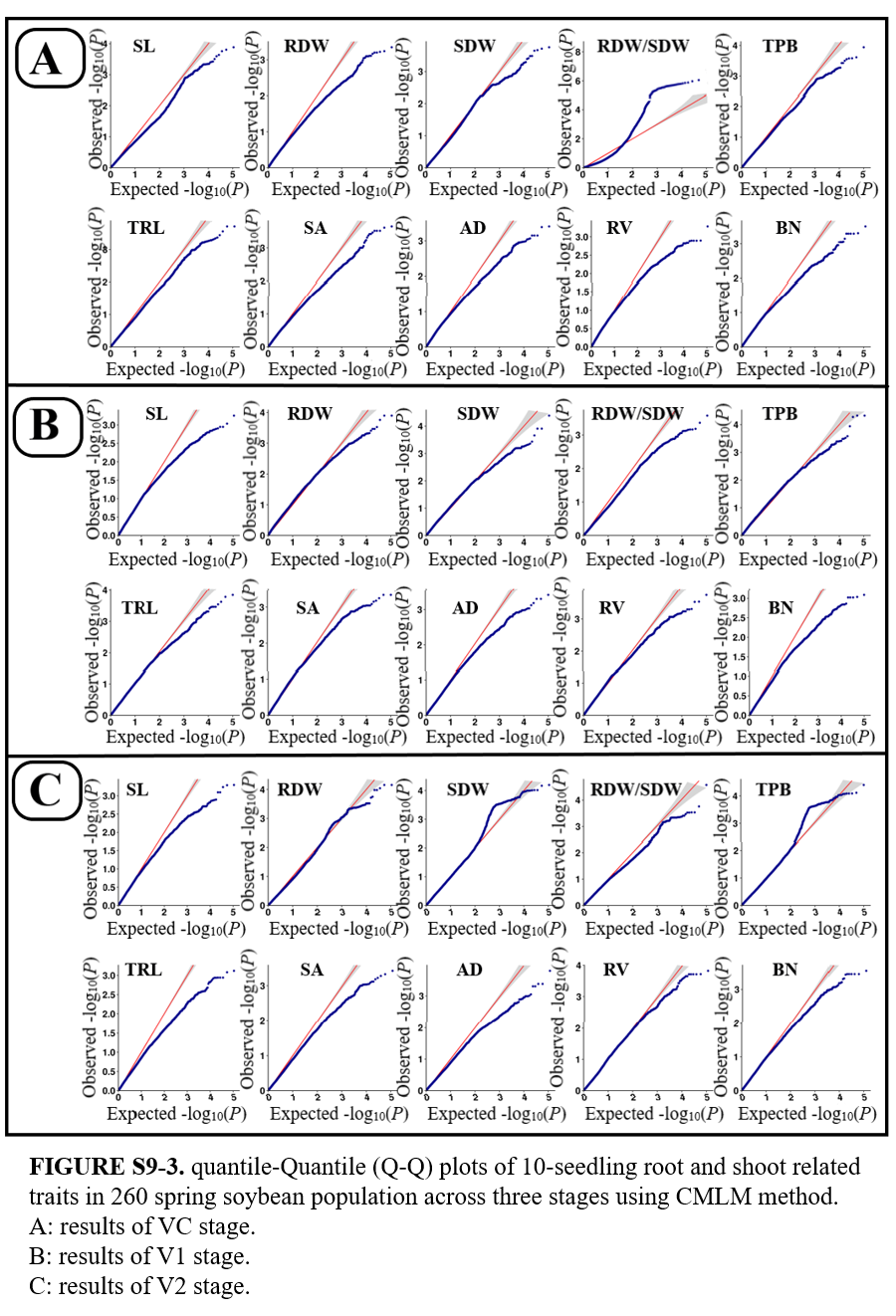

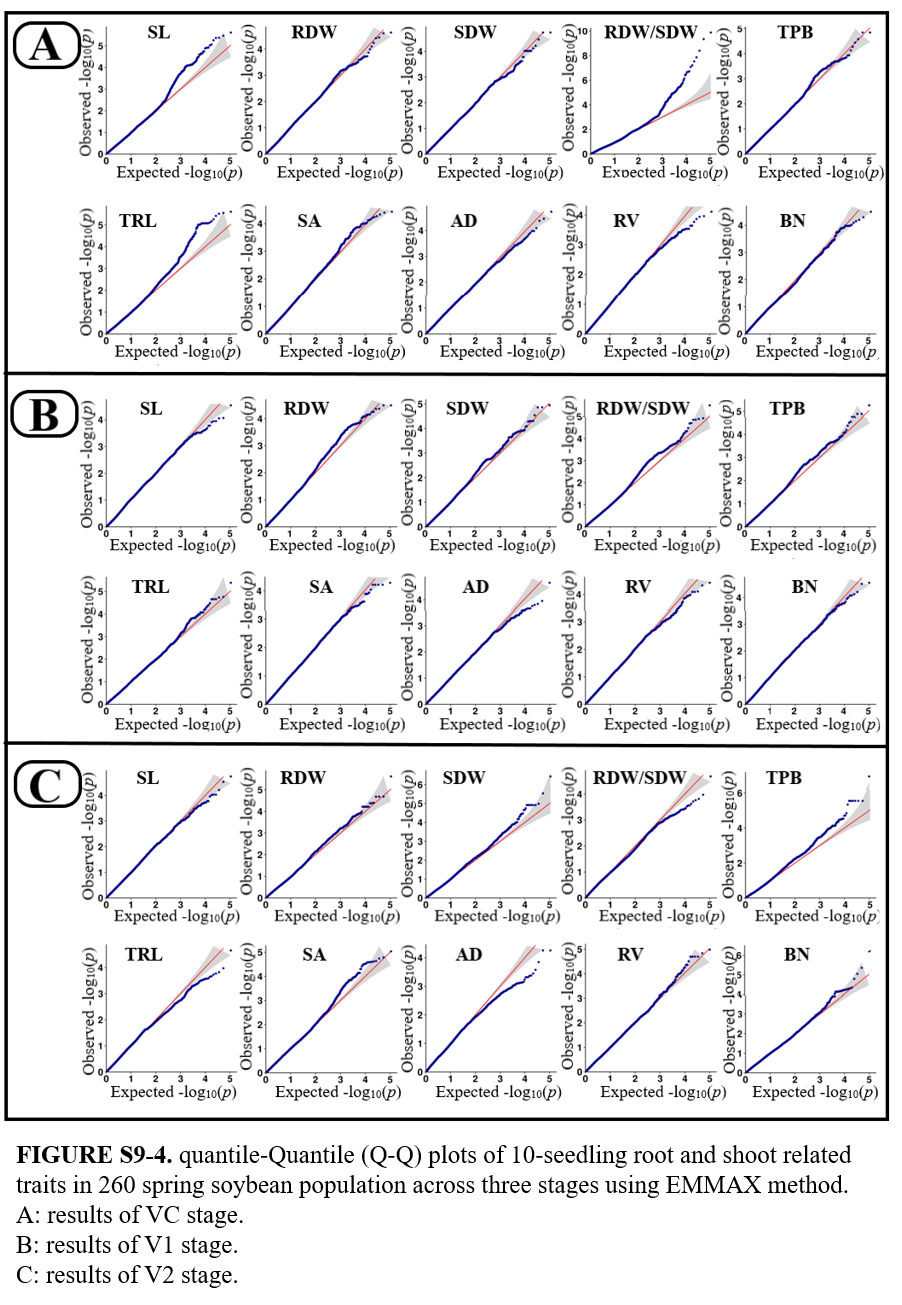

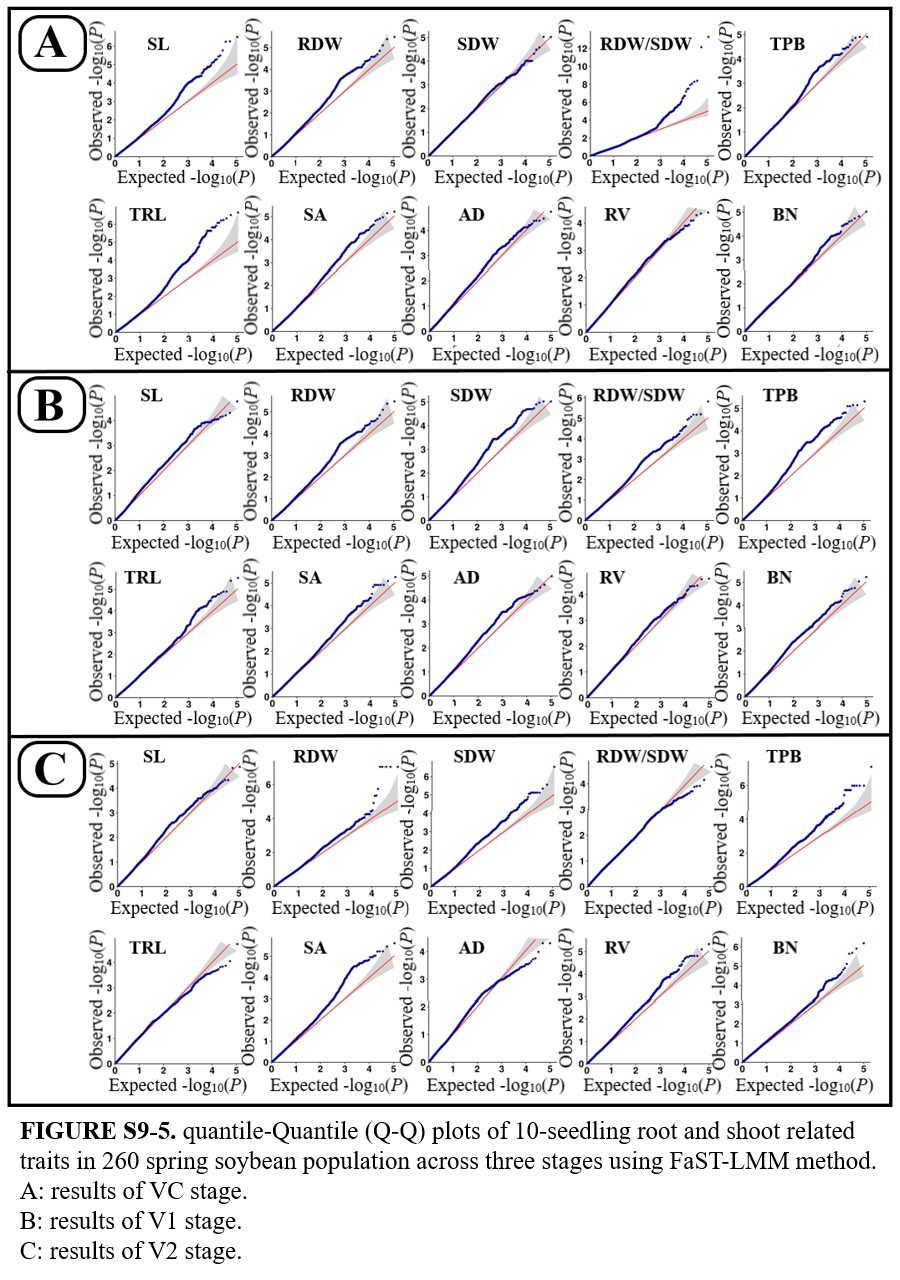

Supplement: Supplementary File 1 — List and provenance of the 260 spring soybean accessions. [file Data_Sheet_1.ZIP › Supplementary File 9.docx]

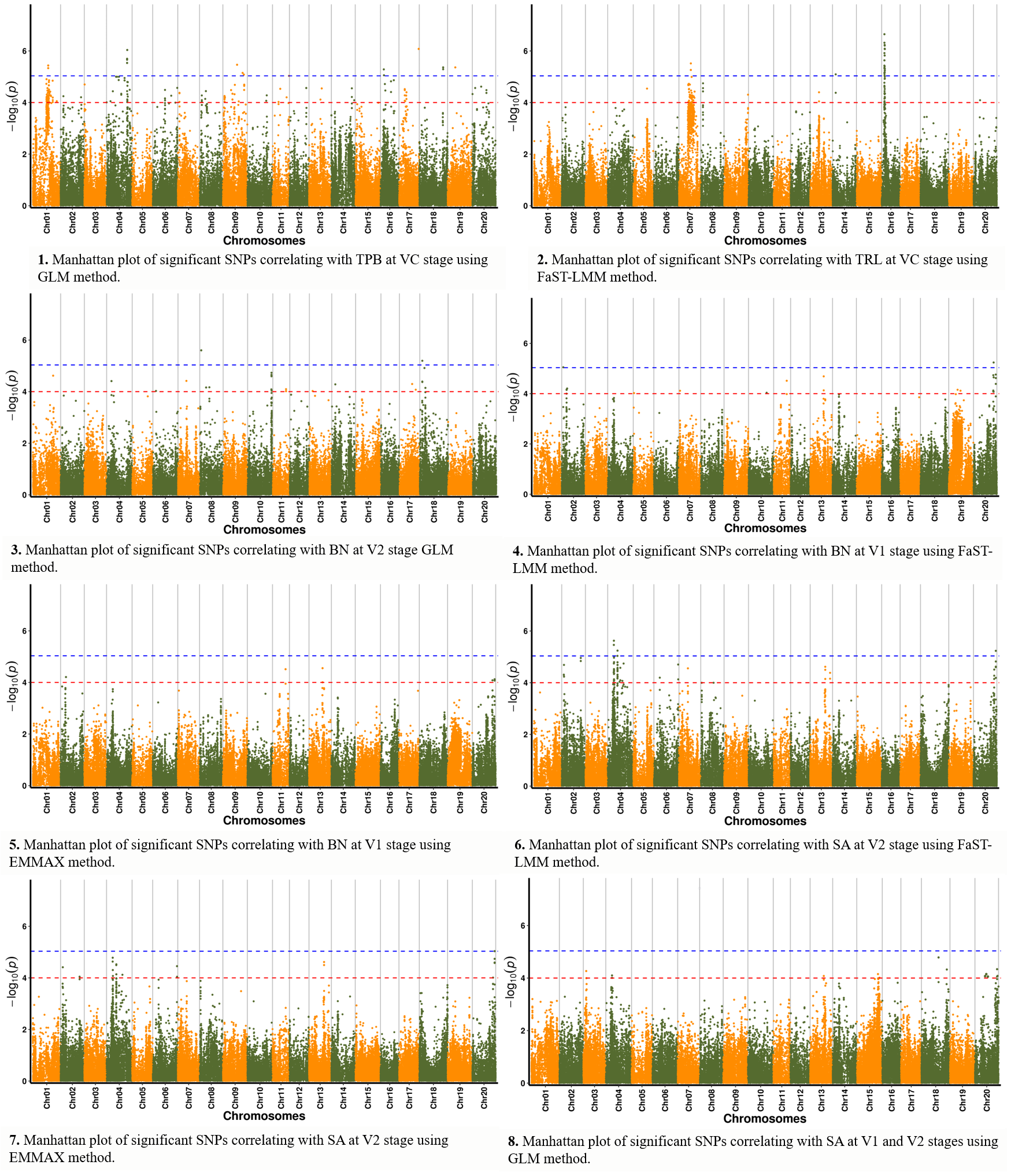


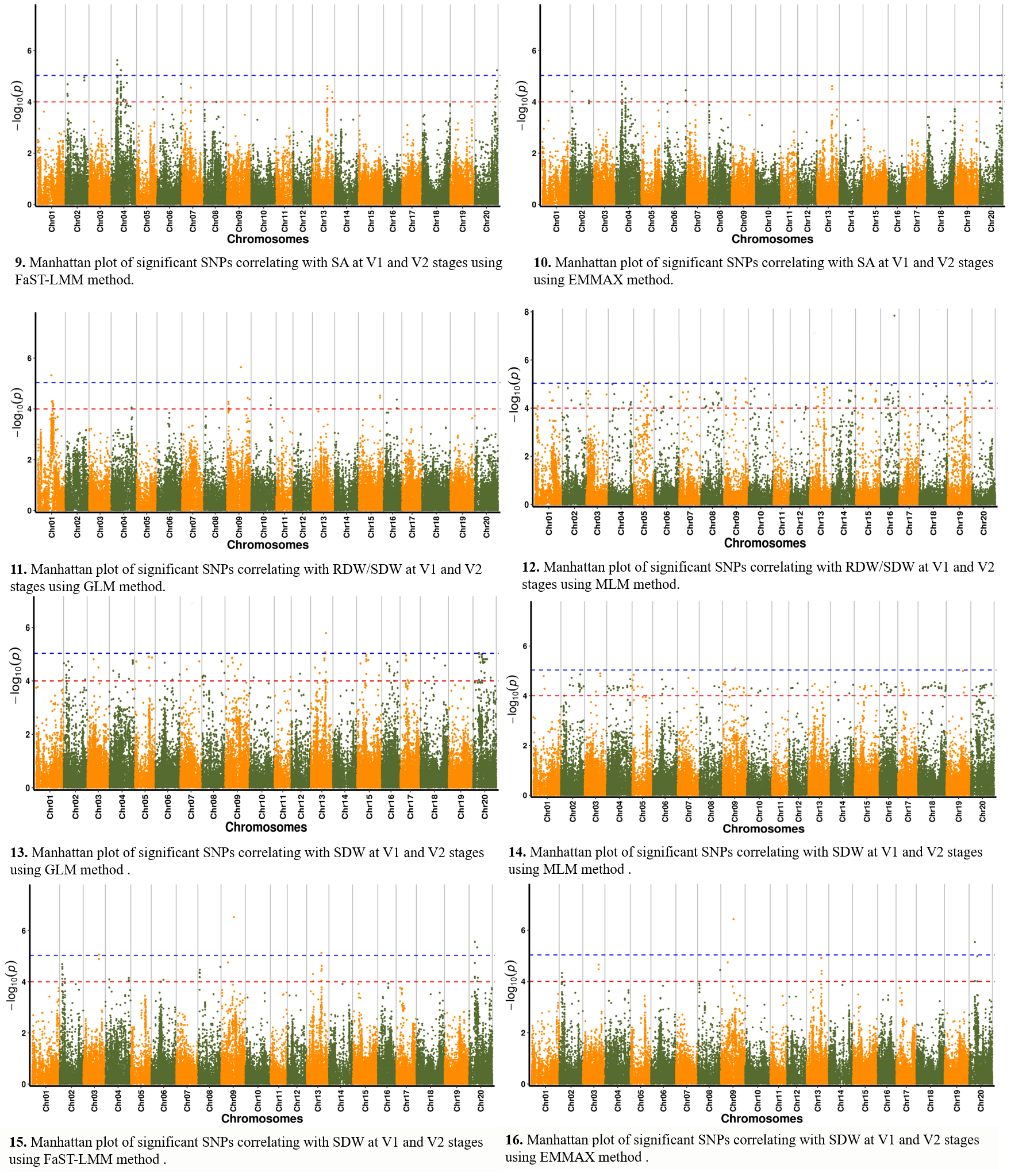


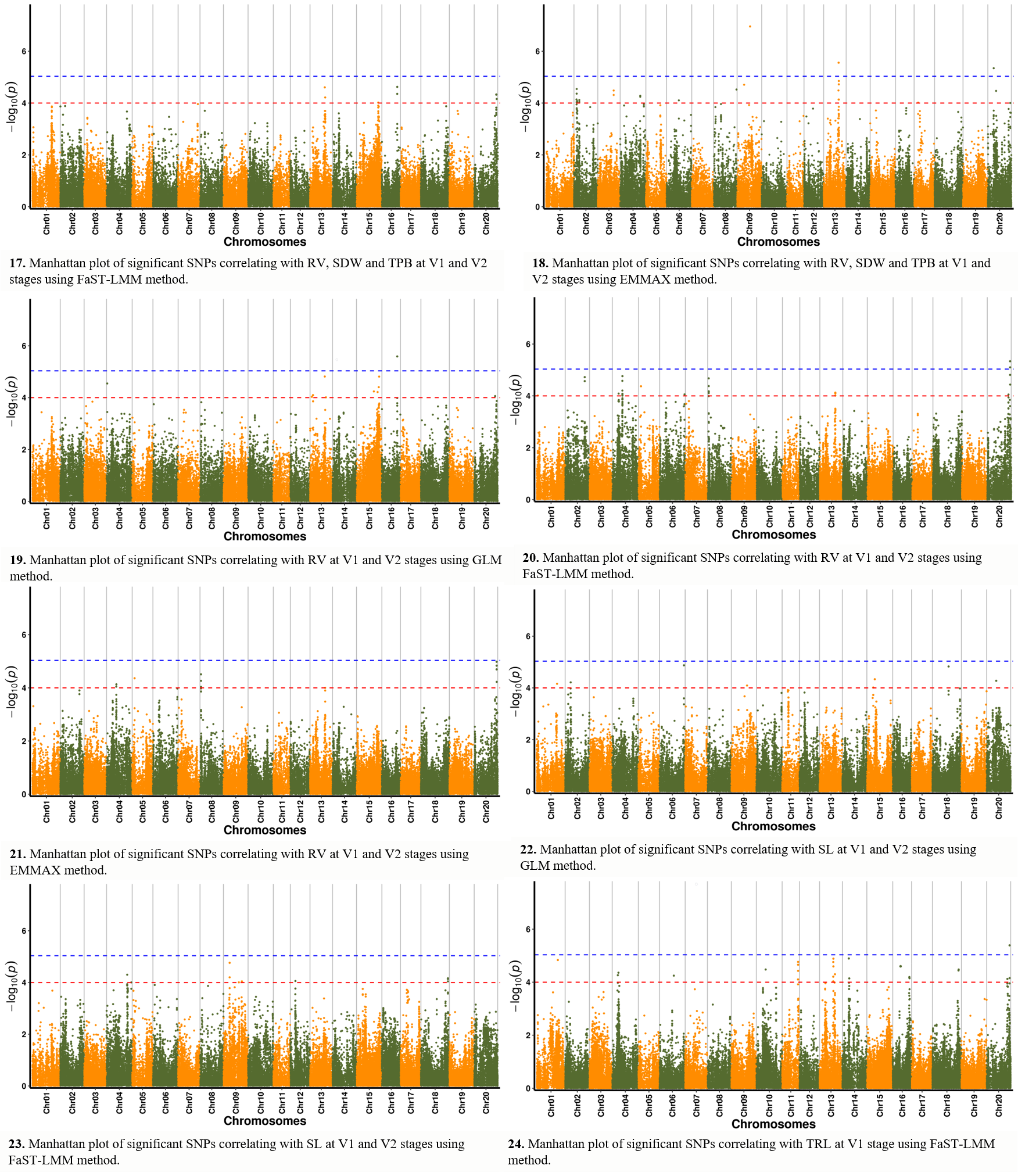


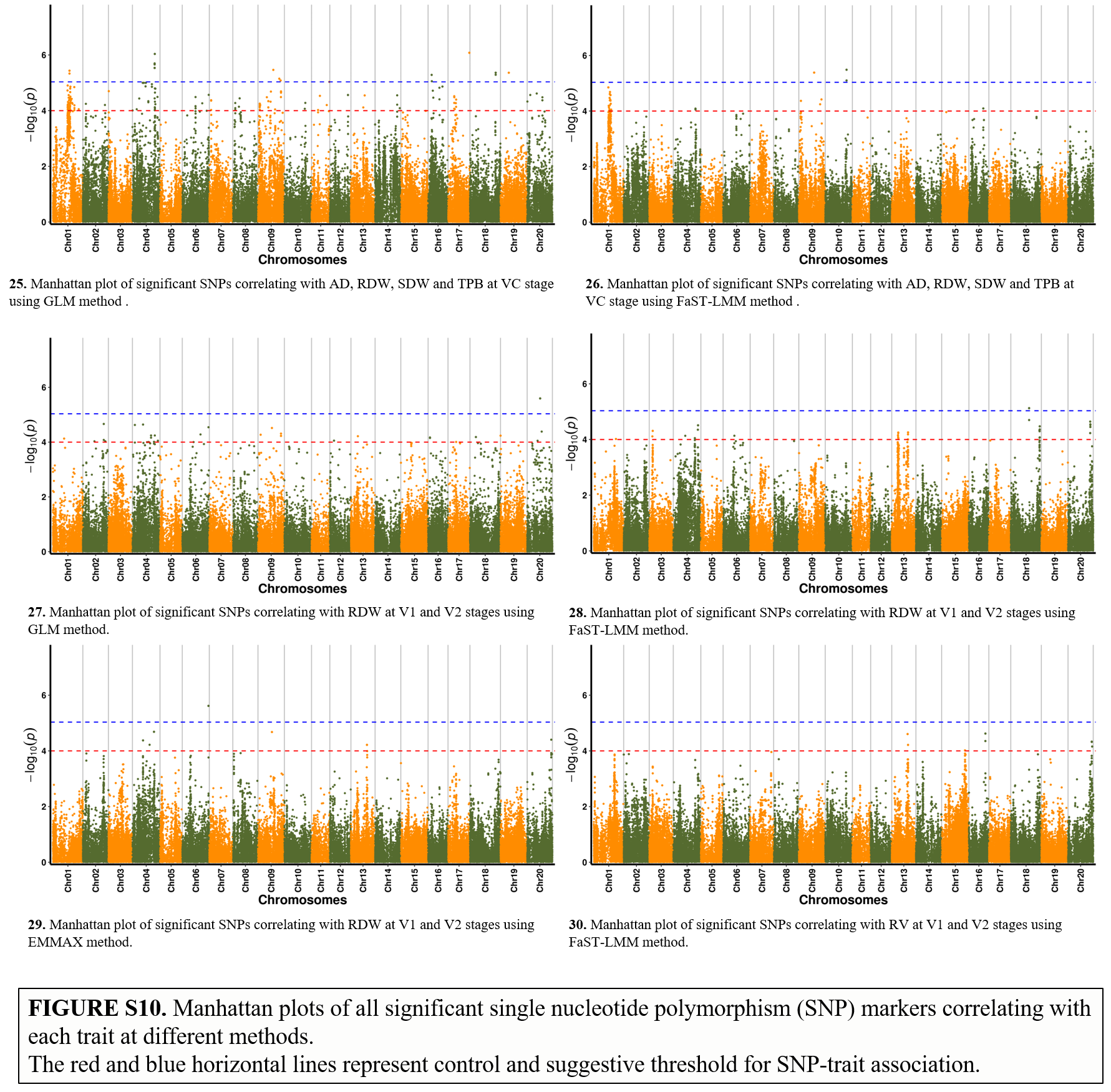

Supplement: Supplementary File 1 — List and provenance of the 260 spring soybean accessions. [file Data_Sheet_1.ZIP › Supplemetary File 10.docx]

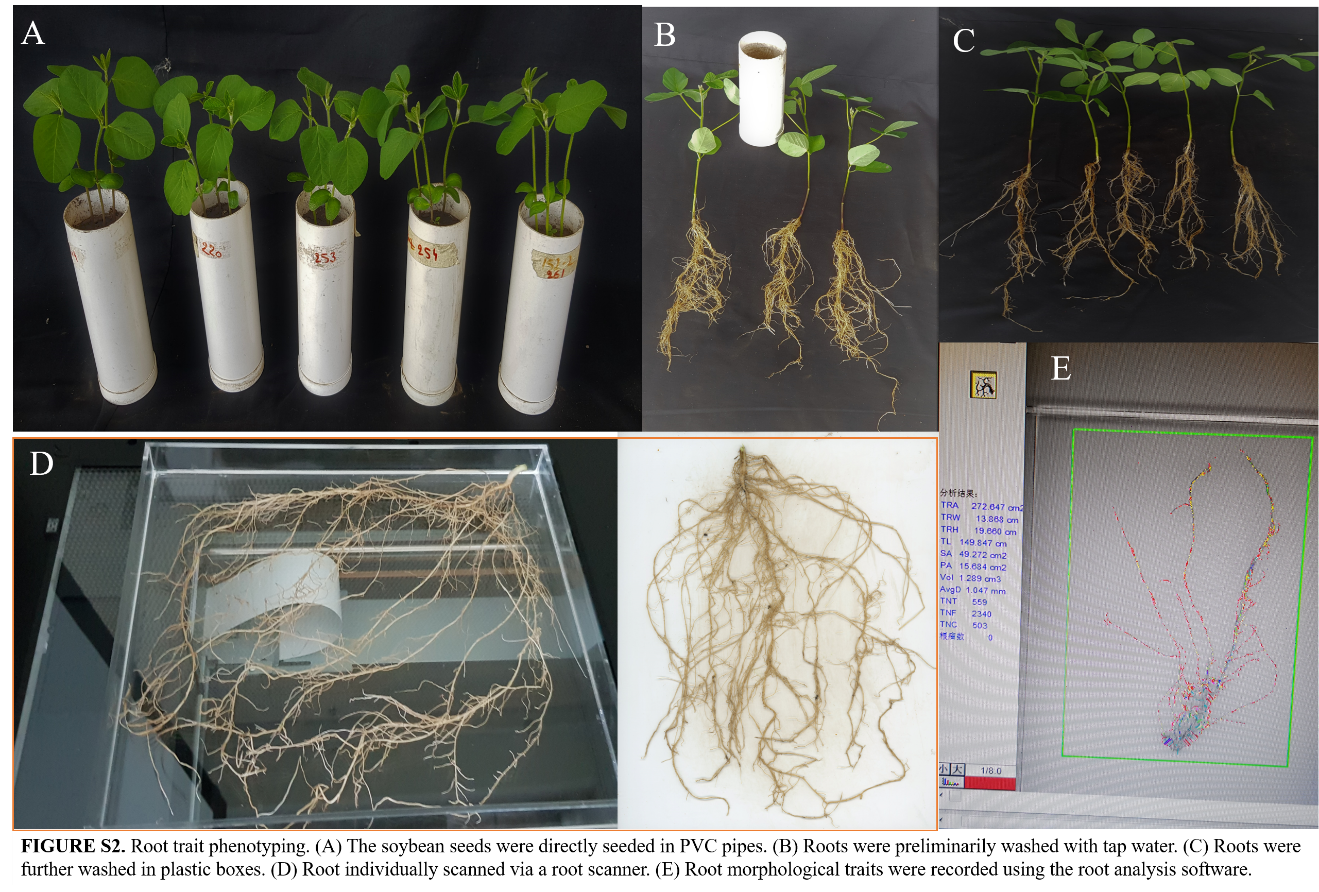

Supplement: Supplementary File 1 — List and provenance of the 260 spring soybean accessions. [file Data_Sheet_1.ZIP › Supplementary File 2.docx]
